# Analysis of Electrocardiographic Criteria of Right Ventricular Hypertrophy in Patients with Chronic Thromboembolic Pulmonary Hypertension before and after Balloon Pulmonary Angioplasty

**DOI:** 10.3390/jcm12134196

**Published:** 2023-06-21

**Authors:** Lukas Ley, Christoph B. Wiedenroth, Hossein Ardeschir Ghofrani, Reinhard Hoeltgen, Dirk Bandorski

**Affiliations:** 1 Campus Kerckhoff, Justus-Liebig-University Giessen, 61231 Bad Nauheim, Germany; lukas.m.ley@med.uni-giessen.de; 2 Kerckhoff Heart and Thorax Center, Department of Thoracic Surgery, 61231 Bad Nauheim, Germany; c.wiedenroth@kerckhoff-klinik.de; 3 Universities of Giessen and Marburg Lung Center (UGMLC), 35392 Giessen, Germany; ardeschir.ghofrani@innere.med.uni-giessen.de; 4 Klinikum Westmünsterland, 46397 Bocholt, Germany; reinhard.hoeltgen@t-online.de; 5 Faculty of Medicine, Semmelweis University Campus Hamburg, 20099 Hamburg, Germany

**Keywords:** PH, pulmonary hypertension, CTEPH, chronic thromboembolic pulmonary hypertension, BPA, balloon pulmonary angioplasty, ECG, electrocardiogram

## Abstract

Background: Chronic thromboembolic pulmonary hypertension (CTEPH) may lead to typical electrocardiographic changes that can be reversed by balloon pulmonary angioplasty (BPA). The aim of this study was to investigate the significance of rarely used electrocardiogram (ECG) parameters, possible electrocardiographic differences between residual and significantly improved CTEPH and the role of electrocardiographic parameters in low mPAP (mean pulmonary arterial pressure) ranges since the mPAP threshold for the definition of pulmonary hypertension has recently been adjusted (≥25 mmHg to >20 mmHg). Material and Methods: Between March 2014 and October 2020, 140 patients with CTEPH and 10 with CTEPD (chronic thromboembolic pulmonary disease) without pulmonary hypertension (PH) were retrospectively enrolled (12-lead ECG and right heart catheterization before and 6 months after BPA). The ECG parameters of right heart strain validated by studies and clinical experience were evaluated. Special attention was paid to six specific ECG parameters. After BPA, the cohort was divided into subgroups to investigate possible electrocardiographic differences with regard to the haemodynamic result. Results: The present study confirmed that the typical electrocardiographic signs of CTEPH can be found on an ECG, can regress after BPA and partially correlate well with haemodynamic parameters. “R V1, V2 + S I, aVL − S V1” was a parameter of particular note. BPA reduced its frequency (47% vs. 29%) statistically significantly after Bonferroni correction (*p* < 0.001). Moreover, it showed a good correlation with mPAP and PVR (r-values: 0.372–0.519, *p*-values: < 0.001). Exceeding its cut-off value before therapy was associated with more severe CTEPH before therapy (higher mPAP, PVR, NT-pro-BNP and troponin and lower TAPSE) and an increased risk of death. Exceeding its cut-off value before and after therapy was associated with more severe CTEPH after therapy (higher RAP, mPAP, PVR, NT-pro-BNP and NYHA class) and an increased risk of death. Men tend to be affected more frequently. After subgrouping, it was observed that a higher median mPAP was associated with a higher right atrial pressure (RAP), a higher pulmonary vascular resistance (PVR) and a lower cardiac output (CO) before and after BPA. In addition, under these conditions, more and more severe electrocardiographic pathologies were detected before and after BPA. Some patients with low mPAP also continued to show mild ECG changes after BPA. In some cases, very few to no pathological ECG changes were detected, and the ECG could present as mostly normal in some patients (5% before BPA and 13% after BPA). Conclusion: “R V1, V2 + S I, aVL − S V1” seems to be able to support the diagnosis of CTEPH, indicate therapeutic improvement and estimate haemodynamics. It also seems capable of predicting a (persistent) severe disease with probably increased need for therapy and increased mortality. Mild PH has been observed to have either no or few mild ECG changes. This might complicate the (early) detection of PH.

## 1. Introduction

Pulmonary hypertension (PH) is a potentially life-threatening cardiovascular disease. PH is classified into five groups based on the underlying pathomechanism [1]. Chronic thromboembolic pulmonary hypertension (CTEPH, group 4) is marked by the chronic obstruction of the pulmonary arteries with a consecutive increase in right ventricular afterload leading to precapillary PH and is a late sequela of acute pulmonary embolism [1,2,3]. The gold standard is the surgical removal of the obstruction via pulmonary endarterectomy. For distal, surgically inaccessible obstructions, balloon pulmonary angioplasty (BPA) is recommended, as well as PH-targeted medical treatment [1,4].

CTEPH is often accompanied by typical electrocardiographic changes, usually due to right ventricular hypertrophy and/or strain. These electrocardiographic findings can contribute to the diagnosis of CTEPH. In addition, an electrocardiogram (ECG) is able to display changes after BPA and thus provide evidence of a response to therapy [5,6,7,8].

The aim of this study was to gain insight into the prevalence of typical ECG changes in CTEPH, their change after BPA and their correlation with haemodynamic parameters. It also aimed to investigate the significance of rarely applied ECG parameters, possible electrocardiographic differences between residual and significantly improved CTEPH and the role of ECG parameters in low mPAP ranges since the mPAP threshold has recently been adjusted.

## 2. Material and Methods

### 2.1. Study Design

This study was conducted as a unicentre, retrospective study in a German referral centre for CTEPH. Consecutive patients who underwent BPA from 11 March 2014 to 19 October 2020 were retrospectively reviewed regarding the inclusion criteria. Patients could be included if they had a confirmed diagnosis of CTEPH according to the 2015 ESC/ERS guideline valid at the start of this study [9], if they had undergone BPA treatment, if sufficient follow-up examination data were available and consent to the study was given. In addition, some patients with a confirmed diagnosis of CTEPD (chronic thromboembolic pulmonary disease) without PH but with severe clinical symptoms, high suffering and mPAP > 20 mmHg were also included. All included patients with CTEPH were also enrolled in the New International CTEPH Database of the International CTEPH Association (NCT02656238). Part of the patient collective (BPA between March 2018 and March 2020) was included in the “International BPA Registry” (NCT03245268), and some patients were part of cohorts covered by previous publications [10,11,12,13,14,15,16,17,18] or were enrolled in ongoing studies within the Collaborative Research Center (CRC1213). An extended positive ethics vote of the Ethics Committee of the Department of Medicine at Justus-Liebig-University dated 17.12.2020 is available (AZ 43/14).

#### Division into Subgroups

To investigate the differences in electrocardiographic characteristics between patients with residual and significantly improved CTEPH, the total cohort was additionally divided into 3 subgroups according to the mPAP level after BPA. The first subgroup had residual CTEPH after BPA according to the 2015 guideline (mPAP ≥ 25 mmHg after BPA, 103 patients) [9], the second subgroup had mild residual CTEPH after BPA according to the current guideline (mPAP = 21–24 mmHg after BPA, 25 patients) [1], and the third subgroup had no residual CTEPH after BPA according to both the 2015 and the current guidelines (mPAP ≤ 20 mmHg after BPA, 19 patients) [1,9].

### 2.2. Electrocardiogram

To evaluate the effect of BPA on electrocardiographic parameters, a twelve-lead ECG was recorded a few days before the first BPA session and 6 months after the last BPA session at follow-up. The twelve-lead ECGs with patients in the supine position were performed by trained technicians and reviewed by two physicians blinded to the clinical characteristics and outcomes of the patients. A commercially available ECG device (MAC 1200 ST, GE Medical Systems, Chicago, IL, USA) was used for the ECG recordings (paper speed: 50 mm/s; sensitivity: 10 mm/mV).

The selection of analysed electrocardiographic parameters corresponds to the typical ECG parameters of right heart strain and hypertrophy; have been used in previous scientific works; and are validated by the American Heart Association, the American College of Cardiology Foundation and the Heart Rhythm Society [5,6,7,8,19,20,21,22,23,24,25,26,27,28,29,30,31,32,33,34].

ECG variables were analysed with standard ECG nomenclature and definitions [35,36]. A complete list of the parameters used can be found in the Supplementary Materials.

#### Main Parameters

Based on our own clinical experience with patients with CTEPH and the results of other clinical studies, the following rarely studied parameters were selected as the main parameters to be focused on in the main analysis:

1. S > R or S > 40 ms in I, II, III ([24]);
2. S > R or S > 40 ms in V6 (own clinical experience);
3. R/S V1 > R/S in V3, V4 ([24]);
4. R/S in V5: R/S in V1 ([8,24]);
5. (RI + SIII) − (SI + RIII) ([8,24]);
6. R V1, V2 + S I, aVL − S V1 ([8,24]).

The parameter “R V1, V2 + S I, aVL − S V1” was calculated as follows: (the highest R-wave amplitude in lead V1 or V2) plus (the highest S-wave amplitude in leads I or aVL) minus S-wave amplitude in lead V1.

### 2.3. Right Heart Catheterization

Right heart catheterization (RHC) was performed according to the standardised Kerckhoff Clinic protocol and the current guidelines to confirm the diagnosis of CTEPH. A 7F Swan–Ganz catheter (Thermodilution Catheter TD1704NX, Bioptimal, Singapore) was advanced into the right heart and pulmonary artery by puncturing the internal jugular vein using Seldinger’s technique. Pressure measurements were made in the right atrium (right atrial pressure: RAP) and the pulmonary artery (mPAP). mPAP was calculated from systolic (sPAP) and diastolic pulmonary artery pressure (dPAP). Cardiac output (CO) was measured using the thermodilution method. Pulmonary vascular resistance (PVR) was calculated according to the following formula:

$$\mathrm{PVR}\;\left({\mathrm{dyn}*\sec*\mathrm{cm}}^{-5}\right)=\frac{\mathrm{mPAP}\;\left(\mathrm{mmHg}\right)-\mathrm{PAWP}\;\left(\mathrm{mmHg}\right)}{\mathrm{CO}\;\left(L/\min\right)}\times 80$$

The parameters used in this study included RAP, mPAP, PVR and CO.

### 2.4. Balloon Pulmonary Angioplasty

BPA was performed as a staged procedure according to the local standard protocol [14,18]. Briefly, central access to the targeted pulmonary segmental artery was established using a sheath and a guiding catheter. Lesions were crossed using a guidewire. Subsequently, vascular obstructions were dilated by balloon inflations. Using selective angiography, improved antegrade flow with good parenchymal perfusion and rapid venous return was interpreted as successful treatment.

### 2.5. Statistics

A statistical analysis was performed by using SPSS Statistics (Version 28, IBM, Armonk, NY, USA) and Jamovi (Version 1.6, Sydney, Australia). Nominal variables are presented as numbers and percentages. Since the Shapiro–Wilk test revealed that almost every variable was non-normal, distributed continuous variables are presented as median and interquartile range. To compare variables before and after the interventional BPA treatment, the McNemar chi-square test for nominal variables and the Wilcoxon signed-rank test for continuous variables were performed. Due to multiple testing, Bonferroni correction had to be applied for the electrocardiographic main criteria. A *p* value < 0.008 was considered to be statistically significant. Spearman‘s rank correlation coefficient was used for a correlation analysis. To compare the results of the group over (>0.6 mV) and the group under (≤0.6 mV) the cut-off value of “R V1, V2 + S I, aVL − S V1“, the chi-square test for nominal variables and the Mann–Whitney-U test for continuous variables were performed.

## 3. Results 

### 3.1. Patient Data

In total, 150 patients, 47.3% male and 52.7% female, with a median age of 63.5 could be included in this study by fulfilling the inclusion criteria (Table 1).

### 3.2. Haemodynamic Data

In total, 140 patients (93%) had CTEPH (mPAP > 25 mmHg) before BPA. In total, 10 patients (7%) with CTEPD without PH (>20 mmHg) were included (Table 2). After BPA, mPAP and PVR decreased noticeably (40 vs. 29 mmHg, 536 vs. 304 dyn∗sec∗cm^−5^, both *p* < 0.001). However, 103 patients (69%) still suffered from residual CTEPH.

### 3.3. Electrocardiographic Data

In the following, all ECG parameters are briefly presented (Table 3 and Table 4). However, the focus is set on the analysis of the main electrocardiographic parameters and subgroups. All ECG data for the entire cohort and the subgroups can be found in detail in the Supplementary Materials.

#### 3.3.1. General Parameters

Before BPA, 99% of the patients were in sinus rhythm (1x atrial fibrillation and 1x atrial flutter). After BPA, 95% were in sinus rhythm (2x atrial fibrillation, 2x atrial flutter and 3x junctional rhythm). The median heart rate was 84 bpm before BPA and decreased to 78 bpm after BPA (*p* < 0.001).

#### 3.3.2. QRS Axis

A decrease in the occurrence of a pathological QRS axis (QRS > 90°, SIQIII type and SISIISIII type) associated with right heart strain was observed after the intervention (56% vs. 32%, *p* < 0.001). A normal QRS axis and left axis deviation occurred more frequently after BPA (35% vs. 53%, *p* < 0.001; 8% vs. 15%, *p* = 0.004).

#### 3.3.3. Atrial Parameters

ECG signs of right atrial strain were considerably less frequent after BPA. Thus, a P dextroatriale was found in 40% of the patients before BPA and only in 16% after BPA (*p* < 0.001). A defined cut-off value (≥0.25 mV) for the P-wave amplitude in lead II was exceeded less often after BPA (32% vs. 11%, *p* < 0.001). Supraventricular extrasystoles (SVESs) occurred infrequently on the ECG both before and after BPA and showed no particular dynamics of improvement (5% vs. 7%, *p* = 0.285).

#### 3.3.4. Ventricular Parameters

Overall, most patients showed improvement in many of the numerously included ventricular ECG parameters, which were representative of a decrease in right ventricular strain and hypertrophy. For example, a defined cut-off value (>1.05) of the parameter “R V1, V2 + S V5, V6” was exceeded less frequently after BPA (45% vs. 27%, *p* < 0.001). Right bundle branch block also occurred less often after the intervention (33% vs. 28%, *p* = 0.074). The defined cut-off value (>1.0) for the height of the R/S ratio in lead V1 was exceeded considerably less frequently after BPA (37% vs. 17%, *p* < 0.001). Ventricular extrasystoles (VESs) occurred infrequently on the ECG both before and after BPA and showed no particular dynamics of improvement (5% vs. 3%, *p* = 0.206).

#### 3.3.5. Repolarisation Parameters

Overall, an improvement in repolarisation disorders was observed after the intervention. Negative T waves in limb leads II, III and aVF (all *p* < 0.001) and precordial leads V1–V3 (*p* = 0.414, *p* = 0.144, *p* < 0.001) were found substantially less frequently after BPA than before. In addition, the QT time (390 ms vs. 380 ms) and QTc time (454 ms vs. 432 ms) were noticeably shorter in median (both *p* < 0.001).

#### 3.3.6. Main Parameters

Overall, the prevalence of the main parameters decreased after the intervention (Table 5). Patients exceeding the cut-off value of “R V1, V2 + S I, aVL − S V1” were observed less frequently after BPA (47% vs. 29%, *p* < 0.001). The same was observed for the parameter “S > R or S > 40 ms in I, II, III” (69% vs. 54%, *p* < 0.001). This was also evident in the parameters “S > R or S > 40 ms in V6” and “R/S V1 > R/S in V3, V4”, but not with the same clarity and frequency. The cut-off value of the parameter “(RI + SIII) − (SI + RIII)” was exceeded before and after BPA in almost all patients (99% vs. 98%, *p* = 0.655), and the cut-off value for the parameter “R/S in V5: R/S in V1” was not exceeded in any patient. After Bonferroni correction, the change in three of the six main parameters was statistically significant.

#### 3.3.7. Sample ECGs

**Figure 1:**
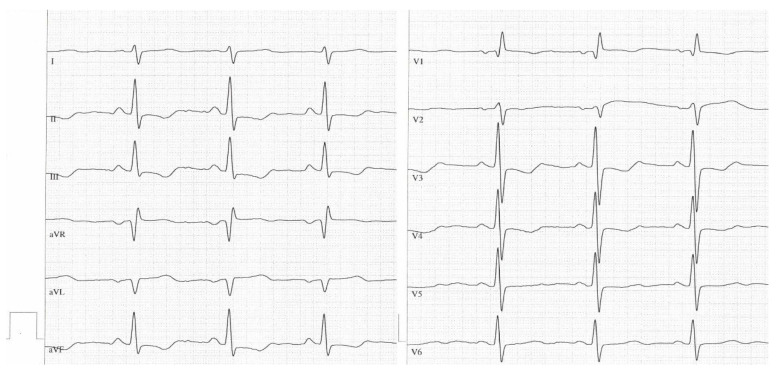
Sample ECG before balloon pulmonary angioplasty. Annotation: A 65-year-old female patient (mPAP: 63 mmHg, PVR: 1073 dyn∗sec∗cm^−5^), QRS axis > 90°, P dextroatriale (P in II > 0.25 mV), right ventricular hypertrophy (Sokolow–Lyon index: 1.6 mV), multiple T-wave inversions (II, III, aVF, V1, V3, V4), R V1, V2 + S I, aVL − S V1 > 0.6 mV (1.05 mV).

**Figure 2:**
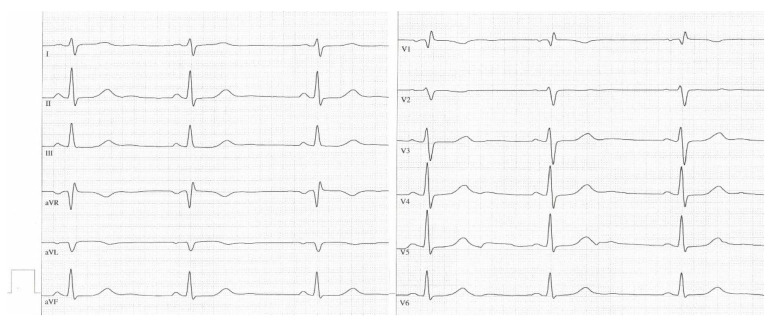
Sample ECG after balloon pulmonary angioplasty. Annotation: A 66-year-old female patient (the same patient, mPAP: 39 mmHg, PVR: 616 dyn∗sec∗cm^−5^), QRS axis > 90°, no P dextroatriale (P in II: 0.2 mV), no right ventricular hypertrophy (Sokolow–Lyon index: 0.7 mV), T-wave inversion only in V1, R V1, V2 + S I, aVL − S V1 ≤ 0.6 mV (0.55 mV).

Figure 1 and Figure 2 are sample ECGs of one patient demonstrating typical ECG signs in CTEPH before BPA and their change after BPA.

### 3.4. Correlation Analysis

**Figure 3:**
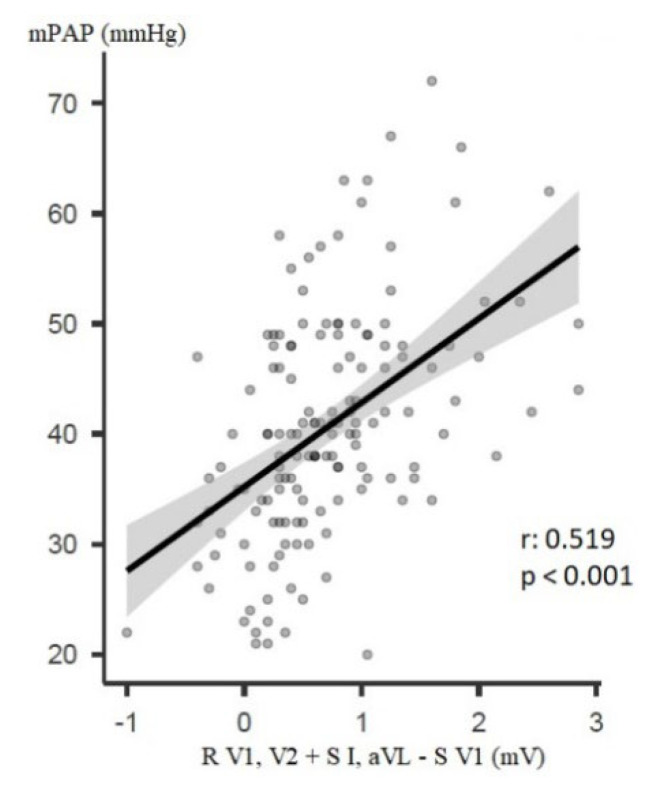
Correlation between “R V1, V2 + S I, aVL − S V1“ and mPAP before BPA. Annotation: mPAP: mean pulmonary artery pressure.

**Figure 4:**
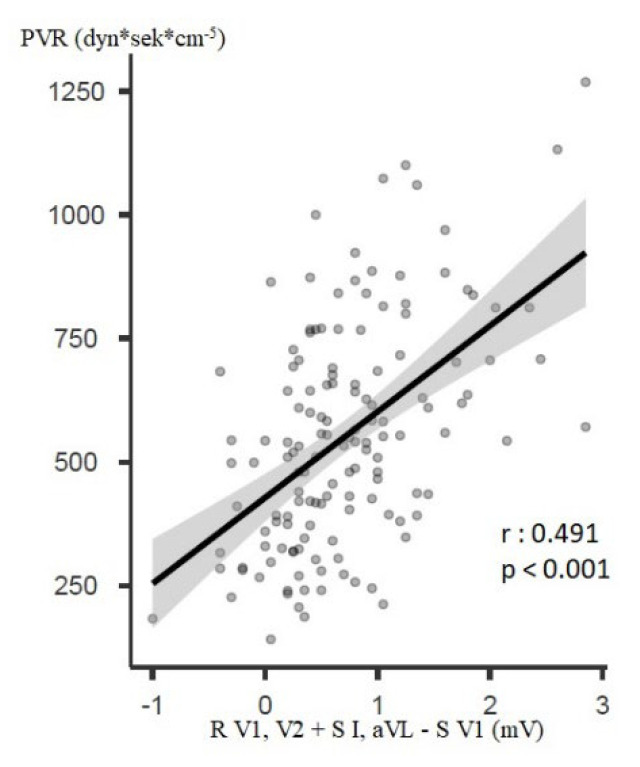
Correlation between “R V1, V2 + S I, aVL − S V1“ and PVR before BPA. Annotation: PVR: pulmonary vascular resistance.

Numerous correlations between the haemodynamic and electrocardiographic parameters of right heart strain could be found. The most important ones (r > 0.5 and *p* < 0.05) are mentioned below. Before BPA, the parameters “R V1, V2 + S I, aVL − S V1” and “R V1, V2 + S I, V6 − S V1” correlated with mPAP (r = 0.524 and r = 0.519, respectively, both *p* < 0.001, Figure 3). The R amplitude in lead V1, the R/S ratio in V1, the parameter “R/S in V5 to R/S in V1” and the parameter “R V1, V2 + S I, V6 − S V1” correlated with PVR (r = 0.522, r = 0.518, r = −0.501 and r = 0.516, all *p* < 0.001). The parameter “R V1, V2 + S I, aVL − S V1” also correlated with PVR (r = 0.491, *p* < 0.001, Figure 4). Further correlations found are shown in the Tables S7–S9 and S21–S23 in the Supplementary Materials.

### 3.5. Subgroup Analysis

There were no age or sex differences detected between the different subgroups. However, haemodynamic differences were observed. The higher the mPAP after BPA, the higher the PVR and RAP and the lower the CO before and after BPA. However, haemodynamics improved similarly in all subgroups.

Identical results were found for the electrocardiographic parameters. The higher the mPAP after BPA, the more and clearer ECG pathologies were detected before and after BPA. Moreover, it was observed that the electrocardiographic parameters generally improved after BPA, regardless of the subgroup. In the correlation analysis, correlations of haemodynamic parameters with electrocardiographic parameters could be determined in all subgroups. Before BPA, e.g., the parameters “R V1, V2 + S I, aVL − S V1”, “R V1, V2 + S I, V6 − S V1” and “R V1 + S V5, V6” could be shown to correlate remarkably with mPAP and PVR in most subgroups. In addition, the change in the parameters “R V1, V2 + S I, aVL − S V1” and “R V1, V2 + S I, V6 − S V1” noticeably correlated with the change in mPAP/PVR in the different subgroups.

### 3.6. Role of the Main Parameter “R V1, V2 + SI, aVL − S V1”

Since the parameter “R V1, V2 + SI, aVL − S V1” often occurred before BPA, improved statistically significantly after BPA, showed strong correlations with mPAP and PVR and has hardly been studied so far, this parameter was analysed in more detail. For this reason, tricuspid annular plane systolic excursion (TAPSE), NT-pro-BNP, troponin, the number of BPA sessions, the number of vessels opened, NYHA stage and the number of deaths were additionally included in the analysis. The cohort was again divided into two groups. This was carried out based on the cut-off value for this parameter (>0.6).

Exceeding the cut-off value before therapy (>0.6 before BPA) was more common in men (58% vs. 37%, *p* = 0.015) and was associated with lower TAPSE (18 vs. 21 mm, *p* = 0.002) before BPA, higher NT-pro-BNP before BPA (1128 vs. 324 pg/mL, *p* < 0.001), higher PVR (615 vs. 431 dyn∗sec∗cm^−5^, *p* < 0.001) and mPAP (43 vs. 36 mmHg, *p* < 0.001) before BPA, higher PVR (337 vs. 269 dyn∗sec∗cm^−5^, *p* = 0.002) and mPAP (31 vs. 25 mmHg, *p* = 0.001) after BPA and more BPA sessions performed (6 vs. 5, *p* < 0.001). In addition, more patients died in this group (12% vs. 2%, *p* = 0.08), and a higher troponin level was observed before BPA (10.3 vs. 8.3 µg/L, *p* = 0.146).

If the cut-off value exceedance persisted after therapy (>0.6 before and after BPA), fewer vessels were opened overall (11 vs. 13, *p* = 0.003); there was a higher RAP (6 vs. 5 mmHg, *p* = 0.009), mPAP (32 vs. 29 mmHg, *p* = 0.007) and PVR (391 vs. 313 dyn∗se∗cm^−5^, *p* = 0.033) after BPA; and a higher NT-pro-BNP level after BPA (254 vs. 87 pg/mL, *p* < 0.001). In addition, more men were represented in this group (64% vs. 50%, *p* = 0.231), more patients died in this group (16% vs. 6%, *p* = 0.292), and a higher NYHA stage remained after therapy (1.8 vs. 1.5, *p* = 0.064).

## 4. Discussion

The present study confirmed that the typical electrocardiographic signs of PH (here CTEPH) can be found on an ECG and regress after therapy (here BPA). A few studies on the electrocardiographic signs of and changes in CTEPH before/after BPA have already been carried out and confirm these results [5,6,7,8]. Further studies in WHO groups 1–4 of PH with various therapies applied came up with comparable results [20,21,22,23,24,25,26,27,28,29,30,31,32,33,34]. In the following discussion, the focus is set on the role of the main electrocardiographic parameters, especially “R V1, V2 + S I, aVL − S V1”, and the role of the different subgroups.

### 4.1. Main Parameters in the Current Literature 

It was observed that a defined cut-off value (<0.04) of the parameter “R/S in V5: R/S in V1” was not undercut by any of the patients both before and after BPA in the present study. This is consistent with the results of Japanese researchers, in whose study none of the patients with CTEPH exceeded this cut-off value before or after BPA either [8]. Other studies reported a prevalence of 2–7% in PAH, CTEPH and IPAH [24,37,38]. Therefore, the assumption arises that the usefulness or the currently used cut-off value for this parameter is limited or not sensitive enough to support the diagnosis of PH.

An “R/S V1 > R/S in V3, V4” was found in 24% of the patients before BPA in the present cohort. Waligora et al. found this pattern in 60% of their PAH cohort, which also included some patients with CTEPH [24]. This observed difference in prevalence could be due to the large proportion of patients with PAH, the poorer mean haemodynamics and thus the higher cardiac workload in Waligora’s study (PVR median in the present study: 536 dyn∗sec∗cm^−5^; PVR mean in Waligora et al.: 1152 dyn∗sec∗cm^−5^) [24]. However, Slawek-Szmyt et al. also found an “R/S V1 > R/S in V3, V4” in 75% of their cohort, although this CTEPH cohort had demographic and haemodynamic characteristics similar to those of the present one [38]. After BPA, the frequency of the occurrence of this parameter was reduced to 10% in this present study (*p* < 0.001). It therefore seems to be able to indicate haemodynamic improvement, but it also seems to appear only in more severe PH. Waligora et al. also found that the parameter occurred less frequently in patients with haemodynamic improvement (65% vs. 56%, *p* = 0.46) [24]. This supports the hypothesis mentioned but was not statistically significant.

Furthermore, it was shown that a defined cut-off value (<1.5 mV) of the parameter “(RI + SIII) − (SI + RIII)” was almost always undershot in CTEPH both before and after BPA (99% vs. 98%; *p* = 0.655). Yokokawa and colleagues reported similar results in their CTEPH cohort before/after BPA (90% vs. 90%, *p* > 0.05) [8]. It is possible that the parameter could be very suitable for suspecting or diagnosing PH but not suitable for monitoring therapy. Moreover, this parameter might be insufficiently specific, or its cut-off value might not be optimal. The results of Waligora et al. and Kopec et al. in patients with PAH, CTEPH and IPAH supported these hypotheses (98% vs. 98%, *p* = 0.99; 96%) [24,37]. Slawek-Szmyt et al. only reported 79% [38].

The parameter “S > R or S > 40 ms in I, II, III” has already been used in a similar but not same way by two other studies [8,38]. Therefore, the results are not directly comparable to ours. Two Polish studies found that “S > R in I” was the most common one (48–55%) [8,38]. An S > R or S > 40 ms in I, II or III was found in the present study in 69% of the patients before BPA and in 54% after BPA (*p* < 0.001). Waligora et al. also observed a reduced frequency after haemodynamic improvement, but this was not statistically significant (76% vs. 57%, *p* = 0.06).

To the best of our knowledge, this is the first study to publish data for the parameter “S > R or S > 40 ms in V6”. This parameter did not occur frequently (23% vs. 19%) nor did it improve strongly or statistically significantly (*p* = 0.157), although it appeared interesting from clinical experience. However, it could not prove this perception in this study.

The role of the parameter “R V1, V2 + S I, aVL − S V1” is described separately.

### 4.2. Role of the Main Parameter “R V1, V2 + S I, aVL − S V1”

The one main parameter of particular note was “R V1, V2 + S I, aVL − S V1”, which proved to be clinically interesting, as many patients (47%) exceeded its cut-off value before BPA. After BPA, this was observed in remarkably fewer patients (29%) and improved statistically significantly after Bonferroni correction (*p* < 0.001). Yokokawa et al. found a similar improvement in its frequency of occurrence in CTEPH and BPA (63% vs. 42%, *p* < 0.05). However, the parameter appeared more often in Yokokawa et al. [8]. This could be explained by the pre-intervention higher mean mPAP (45 vs. 40 mmHg), by gender differences (79% vs. 53% female) and/or by the small number of patients included (n = 19 vs. n = 150). In studies that included mainly patients with PAH and IPAH, the cut-off value of this parameter was exceeded even in 80–83% [24,37]. This suggests that the sensitivity of the parameter might be higher in PAH than in CTEPH. A more detailed exploration by means of further studies is required. The parameter “R V1, V2 + S I, aVL − S V1” thus seems to be able to support the CTEPH diagnosis and indicate therapeutic improvement. It is questionable whether the absolute value of this parameter is important. Although it was improved by BPA in the present study (0.6 mV vs. 0.3 mV; *p* < 0.001), it is difficult to interpret. Moreover, this parameter correlated with mPAP and PVR at baseline and follow-up, and the change in this parameter correlated with the change in mPAP and PVR (r-values: 0.372–0.519, *p*-values: < 0.001) and thus seems to be able to estimate mPAP and PVR.

Exceeding the cut-off value of the parameter “R V1, V2 + S I, aVL − S V1” before therapy was associated with more severe CTEPH haemodynamically (higher mPAP and PVR), echocardiographically (lower TAPSE) and in blood levels (higher NT-pro-BNP and troponin) before therapy. The patients who exceeded the cut-off value had a higher risk of death (12% vs. 2%). As men were more likely to exceed the cut-off value before therapy, men may be more severely ill and more likely to die on average. The patients who exceeded the cut-off value also received more BPA sessions. A possible reason may be more severe disease (older or more complicated occlusions or more occlusions).

If “R V1, V2 + S I, aVL − S V1” exceeded the cut-off value before and after therapy, this was associated with more severe CTEPH after therapy in terms of blood levels (higher NT-pro-BNP) and haemodynamics (higher RAP, mPAP and PVR). These patients also had a higher NYHA class after therapy (1.8 vs. 1.5) and fewer opened vessels overall (11 vs. 13). This could be due to larger, more central lesions or severely altered vessels that cannot be opened. Furthermore, these patients showed a higher risk of death (16% vs. 6%). The parameter “R V1, V2 + S I, aVL − S V1” thus seems to be able to predict a (persistent) severe disease with probably increased need for therapy. It could also support the prediction of mortality or serve as a control variable of successfully performed therapy, as well as an indicator for further therapy. Waligora et al. already reported that a decreased value of the parameter “R V1, V2 + S I, aVL − S V1” predicted an improvement in haemodynamics after therapy in CTEPH and PAH [24]. Moreover, according to these data, men seem to have a more severe disease. This matches with the findings of Asano et al., who found that male gender was an independent predictor of right ventricular dysfunction after BPA [5].

### 4.3. The Role of the Subgroups

The lower the mPAP, the less frequently and less severe electrocardiographic pathologies were observed after BPA. Nevertheless, some patients with low mPAP continued to show mild ECG changes. This could be due to a variety of reasons. On the one hand, residual CTEPH may (still) have been present; on the other hand, the reverse remodelling of the right heart may not yet have been completed. Both theories are supported by the fact that only some ECGs were (already) completely normal at low mPAP after BPA (13%). Approximately 9–13% of ECGs appear to be normal in PH [33,39]. In the present study, 6% of the ECGs before BPA and 13% after BPA were mostly normal.

This observation reveals a crucial problem of the use of an ECG in the diagnosis of (mild) PH. The right ventricle has to hypertrophy two- to three-fold before it accumulates a larger muscle mass than the left ventricle to be “visible” on an ECG [40,41,42]. This usually takes severe right ventricular strain, i.e., an advanced stage of PH [40]. For this reason, electrocardiographic signs are usually rare in mild PH and may be absent altogether [33,39,43,44]. Thus, an ECG can provide clear evidence of PH, especially in severe PH, but the absence of ECG signs does not exclude PH [19,27,32,45,46,47,48].

### 4.4. Strengths and Limitations

The present study can score points for several reasons. It has an acceptably large sample size and a very extensive selection of variables, some of which were used for the first time. In addition, it is, to the best of our knowledge, the first study to evaluate the differences in electrocardiographic changes between the different subgroups of residual or significantly improved CTEPH after BPA. Furthermore, a comprehensive correlation analysis could be presented.

However, there are some limitations to the present study. This study was conducted as a unicentre retrospective study; therefore, possible selection and information bias cannot be excluded. Although 150 patients were included, a significantly higher number of patients and a multicentre approach would be needed for truly reliable conclusions. Moreover, the cohort size after the splitting into subgroups was partly small (103 vs. 25 vs. 19 patients).

Another limitation is the relatively short follow-up period after BPA of 6 months. Thus, it could not be assessed whether the ECG changes were and are permanent and sustained. Moreover, the electrocardiographic changes after BPA were not validated against altered cardiac morphology and function via cardiac imaging (echocardiography or cardiac magnetic resonance imaging). Furthermore, the evaluation of the collected data did not include patient data, e.g., regarding comorbidities, the duration from diagnosis to therapy, the duration of illness and previous or parallel drug treatments. This is a disadvantage, as it has been observed that clinical information can support the evaluation of ECGs for signs of right heart strain [39]. In addition, the duration from diagnosis to therapy or PH medication before and after BPA could have influenced the results. Furthermore, it should be considered that only spontaneous 12-lead ECGs were used in this study, which only allow for an assessment of the electrical cardiac processes in a very limited period of time. Paroxysmal arrhythmias or heart rate variabilities could therefore have been missed. Finally, the analysis was limited to a specific subtype of PH and a highly specific PH therapy. Whether the results can be applied to all subgroups of PH and all PH therapies remains unclear.

## 5. Conclusions

The present study confirmed that the typical electrocardiographic signs of CTEPH can be found on an ECG and regress after haemodynamically successful BPA. It also confirmed that some ECG parameters correlate well with haemodynamic parameters.

After an analysis of the main electrocardiographic parameters, “R V1, V2 + S I, aVL − S V1” stood out. It seems to be able to support the CTEPH diagnosis, indicate a therapeutic improvement and estimate mPAP and PVR. It also seems capable of predicting a (persistent) severe disease with probably increased need for therapy and increased mortality.

Mild PH has been observed to have either no or few mild ECG changes. This might complicate the (early) detection of PH.

## Figures and Tables

**Table 1:** Patient data.

	Baseline	Follow-Up
sex, m/f, *n* (%)	71 (47.3%), 79 (52.7%)	71 (47.3%), 79 (52.7%)
age, years, median (IQR)	63.5 (18.8)	65.3 (18.6)
age, m/f, years, median (IQR)	60.6 (19.9)/65.8 (15.8)	62.6 (20.7)/67.1 (15.8)

Annotation: m: male, f: female, IQR: interquartile range.

**Table 2:** Haemodynamic data.

	Baseline	Follow-Up	*p*
RAP, mmHg, median (IQR)	6 (4)	5 (3)	<0.001
mPAP, mmHg, median (IQR)	40 (13.8)	29 (12)	<0.001
CO, L/min, median (IQR)	4.7 (1.6)	5 (1.4)	0.004
PVR, dyn∗sec∗cm^−5^, median (IQR)	536 (312)	304 (206)	<0.001

Annotation: RAP: right atrial pressure, mPAP: mean pulmonary artery pressure, CO: cardiac output, PVR: pulmonary vascular resistance, IQR: interquartile range.

**Table 3:** ECG parameters.

ECG Parameter	Baseline	Follow-Up	*p*-Value
Sinus rhythm, *n* (%)	148 (99%)	143 (95%)	0.025
Heart rate, bpm, median (IQR)	84 (21.8)	78 (19)	<0.001
QRS axis > 90°, *n* (%)	63 (42%)	34 (23%)	<0.001
QRS axis > 120°, *n* (%)	23 (12%)	10 (7%)	0.002
SISIISIII type, *n* (%)	11 (7%)	12 (8%)	0.705
SIQIII type, *n* (%)	10 (7%)	2 (1%)	0.005
QRS axis associated with right heart strain, *n* (%)	84 (56%)	48 (32%)	<0.001
Left axis deviation, *n* (%)	12 (8%)	22 (15%)	0.004
Normal QRS axis, *n* (%)	53 (35%)	80 (53%)	<0.001
P dextroatriale, *n* (%)	60 (40%)	24 (16%)	<0.001
P biatriale, *n* (%)	10 (7%)	3 (2%)	0.008
P-wave amplitude in II, mV, median (IQR)	0.2 (0.1)	0.2 (0.05)	<0.001
Right ventricular hypertrophy (Sokolow–Lyon index), *n* (%)	67 (45%)	40 (27%)	<0.001
Biventricular hypertrophy (Sokolow–Lyon index), *n* (%)	0 (0%)	1 (1%)	
qR pattern in V1, *n* (%)	24 (16%)	19 (13%)	0.297
Right bundle branch block, *n* (%)	50 (33%)	42 (28%)	0.074
Incomplete right bundle branch block, *n* (%)	28 (19%)	23 (15%)	0.275
Complete right bundle branch block, *n* (%)	22 (15%)	19 (13%)	0.405
R-wave amplitude in V1, mV, median (IQR)	0.3 (0.35)	0.2 (0.25)	<0.001
R-wave amplitude in V2, mV, median (IQR)	0.3 (0.3)	0.25 (0.25)	0.003
S-wave amplitude in V5, mV, median (IQR)	0.5 (0.45)	0.4 (0.4)	<0.001
S-wave amplitude in V6, mV, median (IQR)	0.3 (0.4)	0.2 (0.29)	<0.001
R/S in V1, median (IQR)	1 (2)	0.4 (0.8)	<0.001
R/S in V5, median (IQR)	1.7 (2)	2.4 (2.7)	<0.001
R/S in V6, median (IQR)	2.4 (2.6)	3.3 (4.4)	<0.001
R V1, V2 + S I, V6 − S V1, mV, median (IQR)	0.6 (0.89)	0.28 (0.7)	<0.001
R V1 + S V5, V6, mV, median (IQR)	0.85 (0.73)	0.65 (0.56)	<0.001
R peak time V1 (QRS duration < 120 ms), ms, median (IQR)	50 (30)	43 (40)	<0.001
QT interval, ms, median (IQR)	390 (70)	380 (40)	<0.001
QTc interval (Bazett), ms, median (IQR)	454 (85)	432 (44)	<0.001
T-wave inversion in II, n (%)	46 (31%)	19 (13%)	<0.001
T-wave inversion in III, n (%)	73 (49%)	49 (33%)	<0.001
T-wave inversion in aVF, n (%)	61 (41%)	26 (17%)	<0.001
T-wave inversion in V1, n (%)	130 (87%)	133 (89%)	0.414
T-wave inversion in V2, n (%)	76 (51%)	68 (45%)	0.144
T-wave inversion in V3, n (%)	90 (60%)	66 (44%)	<0.001

Annotation: IQR: interquartile range.

**Table 4:** Cut-off values of ECG parameters.

ECG Parameter	Cut-Off Value	Baseline	Follow-Up	*p*-Value
P-wave amplitude in II, *n* (%)	≥0.25 mV	48 (32%)	17 (11%)	<0.001
R-wave amplitude in V1, *n* (%)	>0.6 mV	22 (15%)	13 (9%)	0.029
S-wave amplitude in V5, *n* (%)	>1.0 mV	13 (9%)	5 (3%)	0.021
S-wave amplitude in V6, n (%)	>0.3 mV	68 (45%)	44 (29%)	<0.001
R/S in V1, *n* (%)	>1	55 (37%)	25 (17%)	<0.001
R/S in V5, *n* (%)	<0.75	18 (12%)	13 (9%)	0.251
R/S in V6, *n* (%)	<0.4	3 (2%)	2 (1%)	0.655
R V1, V2 + S I, V6 − S V1, *n* (%)	>0.6 mV	69 (46%)	40 (27%)	<0.001
R V1 + S V5, V6, *n* (%)	>1.05 mV	48 (32%)	27 (18%)	<0.001
R peak time V1 (QRS duration < 120 ms), *n* (%)	>35 ms	86 (57%)	67 (45%)	0.002

**Table 5:** Cut-off values of electrocardiographic main parameters.

ECG Parameter	Cut-Off Value	Baseline	Follow-Up	*p*-Value
S > R or S > 40 ms in I, II, III, n (%)	positive	104 (69%)	81 (54%)	<0.001 *
S > R or S > 40 ms in V6, n (%)	positive	35 (23%)	29 (19%)	0.157
R/S V1 > R/S V3, V4, n (%)	positive	36 (24%)	15 (10%)	<0.001 *
R/S V5: R/S V1, n (%)	<0.04	0 (0%)	0 (0%)	
(RI + SIII) − (SI + RIII), n (%)	<1.5 mV	148 (99%)	147 (98%)	0.655
R V1, V2 + S I, aVL − S V1, n (%)	>0.6 mV	71 (47%)	44 (29%)	<0.001 *

Annotation: *: *p*-values < 0.008 are considered to be statistically significant after Bonferroni correction.

## Data Availability

The data presented in this study is completely contained within the article or Supplementary Materials.

## Supplementary Materials

The following supporting information can be downloaded at: https://www.mdpi.com/article/10.3390/jcm12134196/s1, Table S1: Electrocardiographic main parameters.; Table S2. Atrial parameters; Table S3: Ventricular parameters; Table S4: Cut-off values of ventricular parameters; Table S5: Repolarisation parameters; Table S6: General electrocardiographic data; Table S7: Correlations between electrocardiographic and haemodynamic parameters—Baseline; Table S8: Correlations between electrocardiographic and haemodynamic parameters—Follow-up; Table S9: Correlations between changes in electrocardiographic and changes in haemodynamic parameters; Table S10: Subgroups patient data; Table S11: Subgroups haemodynamic data; Table S12: Subgroups electrocardiographic main parameters; Table S13: Subgroups electrocardiographic main parameters—cut-off values; Table S14: Subgroups QRS axis; Table S15: Subgroups atrial parameters; Table S16: Subgroups atrial parameters - cut-off values; Table S17: Subgroups ventricular parameters—cut-off values; Table S18: Subgroups ventricular parameters; Table S19: Subgroups repolarisation parameters; Table S20: Subgroups general parameters; Table S21: Subgroups correlations between electrocardiographic and haemodynamic parameters—Baseline; Table S22: Subgroups correlations between electrocardiographic and haemodynamic parameters - Follow-up; Table S23: Subgroups correlations between changes in electrocardiographic and changes in haemodynamic parameters
