# Supplementary material for: Analysis of Electrocardiographic Criteria of Right Ventricular Hypertrophy in Patients with Chronic Thromboembolic Pulmonary Hypertension before and after Balloon Pulmonary Angioplasty
Source: J Clin Med. 2023 Jun 21;12(13):4196. doi: 10.3390/jcm12134196 (PMC10342268; doi:10.3390/jcm12134196)
Supplement: Supplementary file 1 [file jcm-12-04196-s001.zip › jcm-2423634-supplementary.pdf]

Table S1. Electrocardiographic main parameters.

	Baseline	Follow-up	p-value
**S > R or S > 40 ms in I, II, III, n (%)**	104 (69%)	81 (54%)	< 0.001*
**S > R or S > 40 ms in V6, n (%)**	35 (23%)	29 (19%)	0.157
**R/S V1 > R/S in V3, V4, n (%)**	36 (24%)	15 (10%)	< 0.001*
**R/S in V5 : R/S in V1, median (IQR)**	1.9 (4.8)	4.9 (11.4)	< 0.001*
**(RI + SIII) - (SI + RIII), mV, median (IQR)**	-0.65 (0.94)	-0.35 (0.89)	< 0.001*
**R V1, V2 + S I, aVL - S V1, mV, median (IQR)**	0.6 (0.7)	0.3 (0.65)	< 0.001*

Annotation: *: p-values < 0.008 are considered to be statistically significant after Bonferroni correction, IQR: interquartile range

Table S2: Atrial parameters

	Baseline	Follow-up	p-value
**Double-peaked P-wave, n (%)**	20 (13%)	16 (11%)	0.405
**P dextroatriale, n (%)**	60 (40%)	24 (16%)	< 0.001
**P sinistroatriale, n (%)**	2 (1%)	2 (1%)	1.000
**P biatriale, n (%)**	10 (7%)	3 (2%)	0.008
**Supraventricular extrasystoles, n (%)**	7 (5%)	11 (7%)	0.285
**P-wave amplitude in II, mV, median (IQR)**	0.2 (0.1)	0.2 (0.05)	< 0.001
**Highest P-wave, mV, median (IQR)**	0.2 (0.1)	0.2 (0.05)	< 0.001
**Longest P-wave, ms, median (IQR)**	120 (5)	120 (10)	0.079
**Shortest P-wave, ms, median (IQR)**	80 (10)	80 (15)	0.755
**P-wave dispersion, ms, median (IQR)**	40 (13)	40 (15)	0.334

Annotation: IQR: interquartile range

Table S3: Ventricular parameters

	Baseline	Follow-up	p-value
**Right ventricular hypertrophy (SLI), n (%)**	67 (45%)	40 (27%)	< 0.001
**Left ventricular hypertrophy (SLI), n (%)**	0 (0%)	0 (0%)	
**Biventricular hypertrophy (SLI), n (%)**	0 (0%)	1 (1%)	
**qR pattern in V1, n (%)**	24 (16%)	19 (13%)	0.297

**RSR` pattern in V1 (QRS > 120 ms), n (%)**	19 (13%)	15 (10%)	0.317
**Right bundle branch block, n (%)**	50 (33%)	42 (28%)	0.074
**Complete right bundle branch block, n (%)**	22 (15%)	19 (13%)	0.405
**Incomplete right bundle branch block, n (%)**	28 (19%)	23 (15%)	0.275
**Left bundle branch block, n (%)**	4 (3%)	5 (3%)	0.317
**Ventricular extrasystoles, n (%)**	8 (5%)	4 (3%)	0.206
**QRS-interval, ms, Median**	95 (20)	95 (25)	0.224
**R-amplitude in I, mV, median (IQR)**	0.3 (0.29)	0.4 (0.25)	< 0.001
**R-amplitude in III, mV, median (IQR)**	0.8 (0.64)	0.6 (0.5)	< 0.001
**R-amplitude in aVR, mV, median (IQR)**	0.5 (0.3)	0.58 (0.35)	0.038
**R-amplitude in V1, mV, median (IQR)**	0.3 (0.35)	0.2 (0.25)	< 0.001
**R-amplitude in V2, mV, median (IQR)**	0.3 (0.3)	0.25 (0.25)	0.003
**R-amplitude in V6, mV, median (IQR)**	0.8 (0.5)	0.8 (0.5)	0.041
**S-amplitude in I, mV, median (IQR)**	0.31 (0.35)	0.25 (0.25)	< 0.001
**S-amplitude in III, mV, median (IQR)**	0.15 (0.3)	0.1 (0.3)	0.946
**S-amplitude in aVL, mV, median (IQR)**	0.45 (0.5)	0.3 (0.45)	< 0.001
**S-amplitude in V1, mV, median (IQR)**	0.3 (0.35)	0.4 (0.4)	< 0.001
**S-amplitude in V3, mV, median (IQR)**	0.7 (0.64)	0.6 (0.54)	0.045
**S-amplitude in V4, mV, median (IQR)**	0.65 (0.65)	0.5 (0.5)	< 0.001
**S-amplitude in V5, mV, median (IQR)**	0.5 (0.45)	0.4 (0.4)	< 0.001
**S-amplitude in V6, mV, median (IQR)**	0.3 (0.4)	0.2 (0.29)	< 0.001
**R/S in V1, median (IQR)**	1 (2)	0.4 (0.8)	< 0.001
**R/S in V5, median (IQR)**	1.7 (2)	2.4 (2.7)	< 0.001
**R/S in V6, median (IQR)**	2.4 (2.6)	3.3 (4.4)	< 0.001
**R V1,2 + S I, aVL - S V1, mV, median (IQR)**	0.6 (0.7)	0.3 (0.65)	< 0.001
**R V1 + S V5, V6, mV, median (IQR)**	0.85 (0.73)	0.65 (0.56)	< 0.001
**R-amplitude V1 (QRS < 120 ms), mV, median (IQR)**	0.25 (0.3)	0.2 (0.21)	< 0.001
**R peak time V1 (QRS < 120 ms), ms, median (IQR)**	50 (30)	43 (40)	< 0.001
**R V1, V2 + S I, V6 - S V1, mV, median (IQR)**	0.6 (0.89)	0.28 (0.7)	< 0.001

Annotation: IQR: interquartile range, SLI: Sokolow-Lyon-Index for ventricular hypertrophy

Table S4: Cut-off values of ventricular parameters

	Cut-off value	Baseline	Follow-up	p-value
**R-amplitude in I, n (%)**	≤ 0.2 mV	48 (32%)	34 (23%)	0.020
**R-amplitude in aVR, n (%)**	> 0.4 mV	97 (65%)	99 (66%)	0.746
**R-amplitude in V1, n (%)**	> 0.6 mV	22 (15%)	13 (9%)	0.029
**R-amplitude in V6, n (%)**	< 0.3 mV	3 (2%)	5 (3%)	0.414
**S-amplitude in V1, n (%)**	< 0.2 mV	44 (29%)	34 (23%)	0.077
**S-amplitude in V5, n (%)**	> 1.0 mV	13 (9%)	5 (3%)	0.021
**S-amplitude in V6, n (%)**	> 0.3 mV	68 (45%)	44 (29%)	< 0.001
**R/S in V1, n (%)**	> 1.0	55 (37%)	25 (17%)	< 0.001
**R/S in V5, n (%)**	< 0.75	18 (12%)	13 (9%)	0.251
**R/S in V6, n (%)**	< 0.4	3 (2%)	2 (1%)	0.655
**R V1 + S V5, V6, n (%)**	> 1.05 mV	48 (32%)	27 (18%)	< 0.001
**R peak time V1 (QRS < 120 ms), n (%)**	> 35 ms	86 (57%)	67 (45%)	0.002
**R V1, V2 + S I, V6 - S V1, n (%)**	> 0.6 mV	69 (46%)	40 (27%)	< 0.001

Table S5: Repolarisation parameters

	Baseline	Follow-Up	p-value
**Longest T-wave, ms, median (IQR)**	300 (80)	300 (58)	0.044
**Shortest T-wave, ms, median (IQR)**	150 (30)	140 (20)	0.572
**T-wave dispersion, ms, median (IQR)**	140 (80)	140 (58)	0.087
**T-wave inversion in II, n (%)**	46 (31%)	19 (13%)	< 0.001
**T-wave inversion in III, n (%)**	73 (49%)	49 (33%)	< 0.001
**T-wave inversion in aVF, n (%)**	61 (41%)	26 (17%)	< 0.001
**T-wave inversion in V1, n (%)**	130 (87%)	133 (89%)	0.414
**T-wave inversion in V2, n (%)**	76 (51%)	68 (45%)	0.144
**T-wave inversion in V3, n (%)**	90 (60%)	66 (44%)	< 0.001
**T-wave inversions in II, III and aVF, V1-V3**	21 (14%)	9 (6%)	0.011
**T-wave inversions in II, III and aVF**	46 (31%)	18 (12%)	< 0.001

**T-wave-inversions in V1-V3**	68 (45%)	53 (35%)	0.025

Annotation: IQR: interquartile range

Table S6: General electrocardiographic data

	Baseline	Follow-Up	p-value
**Sinusrhythm, n (%)**	148 (99%)	143 (95%)	0.025
**Normocardia, n (%)**	123 (82%)	131 (87%)	0.194
**Bradycardia, n (%)**	3 (2%)	9 (6%)	0.083
**Tachycardia, n (%)**	24 (16%)	10 (7%)	0.006
**First-degree AV block, n (%)**	6 (4%)	4 (3%)	0.564
**Myocardial infarction, n (%)**	6 (4%)	6 (4%)	
**Heart rate, bpm, median (IQR)**	84 (21.8)	78 (19)	< 0.001
**PQ-interval, ms, median (IQR)**	150 (29)	150 (25)	0.454
**PR-interval, ms, median (IQR)**	160 (20)	145 (41)	
**QT-interval, ms, median (IQR)**	390 (70)	380 (40)	< 0.001
**QTc-interval (Bazett), ms, median (IQR)**	454 (85)	432 (44)	< 0.001
**QTc-interval (Fridericia), ms, median (IQR)**	428 (77)	413 (43)	< 0.001
**QTc-interval (Hodges), ms, median (IQR)**	428 (69)	414 (39)	< 0.001
**QTc-interval (Framingham), ms, median (IQR)**	390 (70)	380 (40)	< 0.001

Annotation: IQR: interquartile range

Table S7: Correlations between electrocardiographic and haemodynamic parameters - Baseline

Parameter 1	Parameter 2	r-value	p-value
R-amplitude in V1	mPAP	0.473	< 0.001
S-amplitude in I	mPAP	0.474	< 0.001
S-amplitude in V1	mPAP	-0.401	< 0.001
S-amplitude in V5	mPAP	0.344	< 0.001
S-amplitude in V6	mPAP	0.419	< 0.001
R/S in V1	mPAP	0.464	< 0.001
R/S in V6	mPAP	-0.328	< 0.001

R/S in V5: R/S in V1	mPAP	-0.434	< 0.001
(RI + SIII) - (SI + RIII)	mPAP	-0.331	< 0.001
R V1, V2 + S I, aVL - S V1	mPAP	0.519	< 0.001
R V1, V2 + S I, V6 - S V1	mPAP	0.524	< 0.001
R V1 + S V5, V6	mPAP	0.471	< 0.001
R-amplitude in V1 (QRS < 120 ms)	mPAP	0.458	< 0.001
R peak time in V1 (QRS < 120 ms)	mPAP	0.324	< 0.001
QTc-interval (Bazett)	mPAP	0.328	< 0.001
Highest P-wave	PVR	0.312	< 0.001
R-amplitude in V1	PVR	0.522	< 0.001
S-amplitude in I	PVR	0.372	< 0.001
S-amplitude in V1	PVR	-0.409	< 0.001
S-amplitude in V5	PVR	0.322	< 0.001
S-amplitude in V6	PVR	0.441	< 0.001
R/S in V1	PVR	0.518	< 0.001
R/S in V6	PVR	-0.384	< 0.001
R/S in V5 : R/S in V1	PVR	-0.501	< 0.001
R V1, V2 + S I, aVL - S V1	PVR	0.491	< 0.001
R V1, V2 + S I, V6 - S V1	PVR	0.516	< 0.001
R V1 + S V5, V6	PVR	0.472	< 0.001
R-amplitude in V1 (QRS < 120 ms)	PVR	0.496	< 0.001
R peak time in V1 (QRS < 120 ms)	PVR	0.306	< 0.001
QTc-interval (Bazett)	PVR	0.447	< 0.001
QTc-interval (Fridericia)	PVR	0.384	< 0.001
QTc-interval (Hodges)	PVR	0.419	< 0.001
S-amplitude in V3	RAP	-0.311	< 0.001
S-amplitude in V6	RAP	0.315	< 0.001
R/S in V6	RAP	-0.350	< 0.001
R peak time in V1 (QRS < 120 ms)	RAP	0.304	< 0.001

Annotation: r = 0.30 – 0.49, r = 0.50 – 0.69, mPAP: mean pulmonary artery pressure, PVR: pulmonary vascular resistance, RAP: right atrial pressure

Table S8: Correlations between electrocardiographic and haemodynamic parameters – Follow-up

**Parameter 1**	**Parameter 2**	**r-value**	**p-value**
S-amplitude in I	mPAP	0.356	< 0.001
S-amplitude in V1	mPAP	-0.337	< 0.001
S-amplitude in V6	mPAP	0.408	< 0.001
R/S in V1	mPAP	0.350	< 0.001
R/S in V5 : R/S in V1	mPAP	-0.359	< 0.001
R V1, V2 + S I, aVL - S V1	mPAP	0.407	< 0.001
R V1, V2 + S I, V6 - S V1	mPAP	0.423	< 0.001
R V1 + S V5, V6	mPAP	0.342	< 0.001
QTc-interval (Bazett)	mPAP	0.322	< 0.001
QTc-interval (Hodges)	mPAP	0.318	< 0.001
S-amplitude in I	PVR	0.329	< 0.001
S-amplitude in V1	PVR	-0.344	< 0.001
S-amplitude in V6	PVR	0.394	< 0.001
R/S in V1	PVR	0.335	< 0.001
R/S in V6	PVR	-0.305	< 0.001
R/S in V5 : R/S in V1	PVR	-0.336	< 0.001
R V1, V2 + S I, aVL - S V1	PVR	0.372	< 0.001
R V1, V2 + S I, V6 - S V1	PVR	0.393	< 0.001
QTc-interval (Bazett)	PVR	0.357	< 0.001
QTc-interval (Fridericia)	PVR	0.315	< 0.001

Annotation: r = 0.30 – 0.49, mPAP: mean pulmonary artery pressure, PVR: pulmonary vascular resistance

Table S9: Correlations between changes in electrocardiographic and changes in haemodynamic parameters

**Parameter 1**	**Parameter 2**	**r-value**	**p-value**
P-wave amplitude in II	mPAP	0.379	< 0.001
Highest P-wave	mPAP	0.407	< 0.001
R-amplitude in II	mPAP	0.383	< 0.001

R/S in V6	mPAP	-0.381	< 0.001
R V1, V2 + S I, aVL - S V1	mPAP	0.488	< 0.001
R V1, V2 + S I, V6 - S V1	mPAP	0.482	< 0.001
QTc-interval (Bazett)	mPAP	0.302	< 0.001
P-wave amplitude in II	PVR	0.339	< 0.001
Highest P-wave	PVR	0.397	< 0.001
R-amplitude in V1	PVR	0.422	< 0.001
R-amplitude in V2	PVR	0.335	< 0.001
R-amplitude in V6	PVR	-0.387	< 0.001
S-amplitude in V4	PVR	0.309	< 0.001
S-amplitude in V6	PVR	0.322	< 0.001
R/S in V1	PVR	0.454	< 0.001
R/S in V5	PVR	-0.305	< 0.001
R/S in V6	PVR	-0.419	< 0.001
R/S in V5 : R/S in V1	PVR	-0.357	< 0.001
R V1, V2 + S I, aVL - S V1	PVR	0.418	< 0.001
R V1, V2 + S I, V6 - S V1	PVR	0.494	< 0.001
R V1 + S V5, V6	PVR	0.360	< 0.001
R-amplitude in V1 (QRS < 120 ms)	PVR	0.493	< 0.001
R peak time in V1 (QRS < 120 ms)	PVR	0.306	< 0.001
R-amplitude in V6	CO	0.318	< 0.001
R/S in V1	CO	-0.335	< 0.001

Annotation: r = 0.30 – 0.49, mPAP: mean pulmonary artery pressure, PVR: pulmonary vascular resistance, CO: cardiac output

Table S10: Subgroups patient data

	**Entire cohort**		**mPAP ≥ 25**		**mPAP = 21-24**		**mPAP ≤ 20**	
	**Baseline**	**Follow-up**	**Baseline**	**Follow-up**	**Baseline**	**Follow-up**	**Baseline**	**Follow-up**
**patients, n**	150	150	103	103	25	25	19	19
**sex, male, n**	71	71	48	48	13	13	9	9
**Sex male, %**	47%	47%	47%	47%	52%	52%	47%	47%

**sex, female, n**	79	79	55	55	12	12	10	10
**sex, female, %**	53%	53%	53%	53%	48%	48%	53%	53%
**Age, years, span**	67	67	56	55	58	57	61	61
**Age, years, Minimum - maximum**	15 - 82	16 - 83	26 - 82	28 - 83	21 - 79	23 - 80	15 - 76	16 - 77
**Age, years, median**	63.5	65.3	64	65.6	61.1	62.4	63.5	65
**Age, years, interquartile range**	(18.8)	(18.6)	(17.5)	(17.5)	(21.2)	(21.3)	(18.6)	(18.3)

Annotation: mPAP: mean pulmonary artery pressure

Table S11: Subgroups haemodynamic data

	**Entire cohort**			**mPAP ≥ 25**			**mPAP = 21-24**			**mPAP ≤ 20**		
	**Baseline**	**Follow-up**	**p**	**Baseline**	**Follow-up**	**p**	**Baseline**	**Follow-up**	**p**	**Baseline**	**Follow-up**	**p**
**RAP, mmHg, median**	6	5	***	7	5	***	6	4	*	6	3	**
**RAP, mmHg, interquartile range**	(4)	(3)	***	(5)	(3.8)	***	(3)	(1.3)	*	(3.8)	(1)	**
**mPAP, mmHg, median**	40	29	***	41	32	***	35	23	***	28	19	***
**mPAP, mmHg, interquartile range**	(13.8)	(12)	***	(12)	(11)	***	(9)	(2)	***	(9.5)	(1)	***
**CO, L/min, median**	4.7	5	**	4.6	5	**	4.9	5	-	5.3	5.1	-
**CO, L/min, interquartile range**	(1.6)	(1.4)	**	(1.6)	(1.5)	**	(1.6)	(1.3)	-	(0.7)	(1.1)	-
**PVR, dyn*sek*cm^{-5}, median**	536	304	***	565	369	***	431	241	***	307	164	***
**PVR, dyn*sek*cm^{-5}, interquartile range**	(312)	(206)	***	(283)	(236)	***	(254)	(64)	***	(142)	(53)	***

Annotation: RAP: right atrial pressure, mPAP: mean pulmonary artery pressure, CO: cardiac output, PVR: pulmonary vascular resistance, -: p > 0.05, *:p < 0.05, **: p < 0.01, ***: p < 0.001

Table S12: Subgroups electrocardiographic main parameters

	**Entire cohort**			**mPAP ≥ 25**			**mPAP 21-24**			**mPAP ≤ 20**		
	**Baseline**	**Follow-up**	**p**	**Baseline**	**Follow-up**	**p**	**Baseline**	**Follow-up**	**p**	**Baseline**	**Follow-up**	**p**
**S > R or S > 40 ms in I, II, III, n**	104	81	***#	77	64	**	15	8	*	9	6	-
**S > R or S > 40 ms in I, II, III, %**	69%	54%	***#	75%	62%	**	60%	32%	*	47%	32%	-
**S > R or S > 40 ms in V6, n**	35	29	-	29	23	-	3	3	-	1	1	
**S > R or S > 40 ms in V6, %**	23%	19%	-	28%	22%	-	12%	12%	-	5%	5%	
**R/S V1 > R/S in V3, V4, n**	36	15	***#	30	15	*	5	0		1	0	
**R/S V1 > R/S in V3, V4, %**	24%	10%	***#	29%	15%	*	20%	0%		5%	0%	
**R/S in V5 : R/S in V1, median**	1.9	4.9	***#	1.5	3.6	**#	3	6.1	-	3.7	8	*

**R/S in V5 : R/S in V1, interquartile range**	(4.8)	(11.4)	***#	(2.9)	(8.5)	**#	(8.2)	(14.7)	-	(7.6)	(9.2)	*
**(RI + SIII) - (SI + RIII), mV, median**	-0.65	-0.35	***#	-0.65	-0.4	***#	-0.45	-0.2	**	-0.65	-0.3	-
**(RI + SIII) - (SI + RIII), mV, interquartile range**	(0.94)	(0.89)	***#	(0.93)	(0.88)	***#	(0.8)	(0.75)	**	(1.23)	(0.68)	-
**R V1, V2 + S I, aVL - S V1, mV, median**	0.6	0.3	***#	0.7	0.4	***#	0.43	0.2	**#	0.23	0.05	**#
**R V1, V2 + S I, aVL - S V1, mV, interquartile range**	(0.7)	(0.65)	***#	(0.65)	(0.7)	***#	(0.59)	(0.3)	**#	(0.5)	(0.4)	**#

Annotation: mPAP: mean pulmonary artery pressure. -: p > 0.05, *: p < 0.05, **: p < 0.01, ***: p < 0.001, #: p-values < 0.008 are considered to be statistically significant after Bonferroni correction

Table S13: Subgroups electrocardiographic main parameters – cut-off values

		Entire cohort			mPAP ≥ 25			mPAP = 21-24			mPAP ≤ 20		
	**Cut-off value**	**Base-line**	**Follow-up**	**p**	**Baseline**	**Follow-up**	**p**	**Baseline**	**Follow-up**	**p**	**Baseline**	**Follow-up**	**p**
**S > R or S > 40 ms in I, II, III, n**	Positive	104	81	***#	77	64	**	15	8	*	9	6	-
**S > R or S > 40 ms in I, II, III, %**	Positive	69%	54%	***#	75%	62%	**	60%	32%	*	47%	32%	-
**S > R or S > 40 ms in V6, n**	Positive	35	29	-	29	23	-	3	3	-	1	1	
**S > R or S > 40 ms in V6, %**	Positive	23%	19%	-	28%	22%	-	12%	12%	-	5%	5%	
**R/S V1 > R/S in V3 or V4, n**	Positive	36	15	***#	30	15	*	5	0		1	0	
**R/S V1 > R/S in V3 or V4, %**	Positive	24%	10%	***#	29%	15%	*	20%	0%		5%	0%	
**R/S in V5 : R/S in V1, n**	< 0.04	0	0		0	0		0	0		0	0	
**R/S in V5 : R/S in V1, %**	< 0.04	0%	0%		0%	0%		0%	0%		0%	0%	
**(RI + SIII) - (SI + RIII), n**	< 1.5 mV	148	147	-	103	101		25	25		17	18	-
**(RI + SIII) - (SI + RIII), %**	< 1.5 mV	99%	98%	-	100%	98%		100%	100%		90%	95%	-
**R V1, V2 + S I, aVL - S V1, n**	> 0.6 mV	71	44	***#	56	39	***#	8	2	*	4	0	
**R V1, V2 + S I, aVL - S V1, %**	> 0.6 mV	47%	29%	***#	54%	38%	***#	32%	8%	*	21%	0%	

Annotation: mPAP: mean pulmonary artery pressure, -: p > 0.05, *: p < 0.05, **: p < 0.01, ***: p < 0.001, #: p-values < 0.008 are considered to be statistically significant after Bonferroni correction

Table S14: Subgroups QRS axis

	Entire cohort			mPAP ≥ 25			mPAP = 21-24			mPAP ≤ 20		
	**Baseline**	**Follow-up**	**p**	**Baseline**	**Follow-up**	**p**	**Baseline**	**Follow-up**	**p**	**Baseline**	**Follow-up**	**p**
**QRS axis > 90°, n**	63	34	< 0.001	49	26	< 0.001	9	4	0.059	3	2	0.564
**QRS axis > 90°, %**	42%	23%	< 0.001	48%	25%	< 0.001	36%	16%	0.059	16%	11%	0.564
**QRS axis > 120°, n**	23	10	0.002	20	7	< 0.001	2	1	0.317	0	1	

**QRS axis > 120°, %**	15%	7%	0.002	19%	7%	< 0.001	8%	4%	0.317	0%	5%	
**SISIISIII pattern, n**	11	12	0.705	9	11	0.317	0	0		2	1	0.564
**SISIISIII pattern, %**	7%	8%	0.705	9%	11%	0.317	0%	0%		11%	5%	0.564
**SIQIII pattern, n**	10	2	0.005	5	0		2	1	0.317	2	0	
**SIQIII pattern, %**	7%	1%	0.005	5%	0%		8%	4%	0.317	11%	0%	
**QRS axis associated with right heart strain, n**	84	48	< 0.001	63	37	< 0.001	11	5	0.034	7	3	0.046
**QRS axis associated with right heart strain, %**	56%	32%	< 0.001	61%	36%	< 0.001	44%	20%	0.034	37%	16%	0.046
**Left axis deviation, n**	12	22	0.004	8	16	0.011	2	4	0.157	2	2	
**Left axis deviation, %**	8%	15%	0.004	8%	16%	0.011	8%	16%	0.157	11%	11%	
**Normal axis, n**	53	80	< 0.001	32	50	< 0.001	12	16	0.206	10	14	0.046
**Normal axis, %**	35%	53%	< 0.001	31%	49%	< 0.001	48%	64%	0.206	53%	74%	0.046

Annotation: mPAP: mean pulmonary artery pressure

Table S15: Subgroups atrial parameters

	**Entire cohort**			**mPAP ≥ 25**			**mPAP = 21-24**			**mPAP ≤ 20**		
	**Baseline**	**Follow-up**	**p**	**Baseline**	**Follow-up**	**p**	**Baseline**	**Follow-up**	**p**	**Baseline**	**Follow-up**	**p**
**Double-peaked P-wave, n**	20	16	-	14	13	-	5	2	-	1	1	
**Double-peaked P-wave, %**	13%	11%	-	14%	13%	-	20%	8%	-	5%	5%	
**P dextroatriale, n**	60	24	***	45	19	***	7	2	*	6	1	*
**P dextroatriale, %**	40%	16%	***	44%	19%	***	29%	8%	*	32%	5%	*
**P sinistroatriale, n**	2	2	-	2	2	-	0	0		0	0	
**P sinistroatriale, %**	1%	1%	-	2%	2%	-	0%	0%		0%	0%	
**P biatriale, n**	10	3	**	6	2	*	3	0		0	0	
**P biatriale, %**	7%	2%	**	6%	2%	*	12%	0%		0%	0%	
**Supraventricular extrasystoles, n**	7	11	-	4	11	*	0	0		3	0	
**Supraventricular extrasystoles, %**	5%	7%	-	4%	11%	*	0%	0%		16%	0%	
**P-wave amplitude in II, mV, median**	0.2	0.2	***	0.2	0.2	***	0.2	0.2	**	0.2	0.15	**
**P-wave amplitude in II, mV, interquartile range**	(0.1)	(0.05)	***	(0.1)	(0.05)	***	(0.08)	(0.1)	**	(0.13)	(0.1)	**

**Highest P-wave, mV, median**	0.2	0.2	***	0.2	0.2	***	0.2	0.2	**	0.2	0.15	**
**Highest P-wave, mV, interquartile range**	(0.1)	(0.05)	***	(0.1)	(0.05)	***	(0.08)	(0.1)	**	(0.13)	(0.1)	**
**Longest P-wave, ms, median**	120	120	*	120	120	-	120	120	-	120	110	*
**Longest P-wave, ms, interquartile range**	(5)	(10)	*	(0)	(20)	-	(11)	(13)	-	(10)	(20)	*
**Shortest P-wave, ms, median**	80	80	-	80	80	-	70	75	-	80	80	-
**Shortest P-wave, ms, interquartile range**	(10)	(15)	-	(15)	(20)	-	(20)	(20)	-	(20)	(20)	-
**P-wave dispersion, ms, median**	40	40	-	40	40	-	40	40	-	40	40	-
**P-wave dispersion, ms, interquartile range**	(13)	(15)	-	(10)	(10)	-	(26)	(5)	-	(20)	(35)	-

Annotation: mPAP: mean pulmonary artery pressure, -: p > 0.05, *: p < 0.05, **: p < 0.01, ***: p < 0.001

Table S16: Subgroups atrial parameters - cut-off values

		**Entire cohort**			**mPAP ≥ 25**			**mPAP = 21-24**			**mPAP ≤ 20**		
	**Cut-off value**	**Baseline**	**Follow-up**	**p**	**Baseline**	**Follow-up**	**p**	**Baseline**	**Follow-up**	**p**	**Baseline**	**Follow-up**	**p**
**P-wave amplitude in II, n**	≥ 0,25 mV	48	17	***	36	12	***	6	1	*	5	1	*
**P-wave amplitude in II, %**	≥ 0,25 mV	32%	11%	***	35%	12%	***	24%	4%	*	26%	5%	*

Annotation: mPAP: mean pulmonary artery pressure, -: p > 0.05, *: p < 0.05, **: p < 0.01, ***: p < 0.001

Table S17: Subgroups ventricular parameters – cut-off values

		**Entire cohort**			**mPAP ≥ 25**			**mPAP = 21-24**			**mPAP ≤20**		
	**Cut-off value**	**Baseline**	**Follow-up**	**p**	**Baseline**	**Follow-up**	**p**	**Baseline**	**Follow-up**	**p**	**Baseline**	**Follow-up**	**p**
**R-amplitude in I, n**	≤ 0,2 mV	48	34	*	31	23	-	8	5	-	9	4	-
**R-amplitude in I, %**	≤ 0,2 mV	32%	23%	*	30%	22%	-	32%	20%	-	47%	21%	-
**R-amplitude in aVR, n**	> 0,4 mV	97	99	-	63	63	-	18	21	-	15	14	-
**R-amplitude in aVR, %**	> 0,4 mV	65%	66%	-	61%	61%	-	72%	84%	-	79%	74%	-
**R-amplitude in V1, n**	> 0,6 mV	22	13	*	18	10	*	3	1	-	0	0	
**R-amplitude in V1, %**	> 0,6 mV	15%	9%	*	18%	10%	*	12%	4%	-	0%	0%	
**R-amplitude in V6, n**	< 0,3 mV	3	5	-	2	4	-	0	1		1	0	
**R-amplitude in V6, %**	< 0,3 mV	2%	3%	-	2%	4%	-	0%	4%		5%	0%	
**S-amplitude in V1, n**	< 0,2 mV	44	34	*	36	30	-	4	2	-	2	1	-
**S-amplitude in V1, %**	< 0,2 mV	29%	23%	*	35%	29%	-	16%	8%	-	11%	5%	-

**S-amplitude in V5, n**	> 1,0 mV	13	5	*	12	5	*	1	0		0	0	
**S-amplitude in V5, %**	> 1,0 mV	9%	3%	*	12%	5%	*	4%	0%		0	0%	
**S-amplitude in V6, n**	> 0,3 mV	68	44	***	51	38	*	10	3	**	5	1	*
**S-amplitude in V6, %**	> 0,3 mV	45%	29%	***	50%	37%	*	40%	12%	**	26%	5%	*
**R/S in V1, n**	> 1,0	55	25	***	46	22	***	7	2	*	1	0	
**R/S in V1, %**	> 1,0	37%	17%	***	45%	21%	***	28%	4%	*	5%	0%	
**R/S in V5, n**	< 0,75	18	13	-	10	9	-	5	2	-	2	1	-
**R/S in V5, %**	< 0,75	12%	9%	-	10%	9%	-	20%	8%	-	11%	5%	-
**R/S in V6, n**	< 0,4	3	2	-	2	2	-	0	0		1	0	
**R/S in V6, %**	< 0,4	2%	1%	-	2%	2%	-	0%	0%		5%	0%	
**R V1 + S V5, V6, n**	> 1,05 mV	48	27	***	38	22	**	7	2	-	1	1	-
**R V1 + S V5, V6, %**	> 1,05 mV	32%	18%	***	37%	21%	**	28%	8%	-	5%	5%	-
**R peak time V1 (QRS < 120 ms), n**	> 35 ms	86	67	**	65	52	*	14	9	-	5	4	-
**R peak time V1 (QRS < 120 ms), %**	> 35 ms	57%	45%	**	63%	51%	*	56%	36%	-	26%	21%	-
**R V1, V2 + S I, V6 - S V1, n**	> 0,6 mV	71	44	***	56	39	***	8	2	*	4	0	
**R V1, V2 + S I, V6 - S V1, %**	> 0,6 mV	47%	29%	***	54%	38%	***	32%	8%	*	21%	0%	

Annotation: mPAP: mean pulmonary artery pressure, -: p > 0.05, *: p < 0.05, **: p < 0.01, ***: p < 0.001

Table S18: Subgroups ventricular parameters

	**Entire cohort**			**mPAP ≥ 25**			**mPAP = 21-24**			**mPAP ≤ 20**		
	**Baseline**	**Follow-up**	**p**	**Baseline**	**Follow-up**	**p**	**Baseline**	**Follow-up**	**p**	**Baseline**	**Follow-up**	**p**
**Right ventricular hypertrophy (SLI), n**	67	40	***	50	33	**	10	3	*	5	2	-
**Right ventricular hypertrophy (SLI), %**	45%	27%	***	49%	32%	**	40%	12%	*	26%	11%	-
**Left ventricular hypertrophy (SLI), n**	0	0		0	0		0	0		0	0	
**Left ventricular hypertrophy (SLI), %**	0%	0%		0%	0%		0%	0%		0%	0%	
**Biventricular hypertrophy (SLI), n**	0	1		0	1		0	0		0	0	
**Biventricular hypertrophy (SLI), %**	0%	1%		0%	1%		0%	0%		0%	0%	
**qR pattern in V1, n**	24	19	-	17	16	-	3	2	-	2	0	
**qR pattern in V1, %**	16%	13%	-	17%	16%	-	12%	8%	-	11%	0%	
**RSR` in V1 (QRS > 120 ms), n**	19	15	-	15	12	-	2	2		1	0	

**RSR` in V1 (QRS > 120 ms), %**	13%	10%	-	15%	12%	-	8%	8%		5%	0%	
**Right bundle branch block, n**	50	42	-	40	33	-	6	5	-	3	3	-
**Right bundle branch block, %**	34%	28%	-	39%	32%	-	24%	20%	-	16%	16%	-
**Complete right bundle branch block, n**	22	19	-	18	16	-	2	2		1	0	
**Complete right bundle branch block, %**	15%	13%	-	18%	16%	-	8%	8%		5%	0%	
**Incomplete right bundle branch block, n**	28	23	-	22	17	-	4	3	-	2	3	-
**Incomplete right bundle branch block, %**	19%	15%	-	21%	17%	-	16%	12%	-	11%	16%	-
**Left bundle branch block, n**	4	5	-	2	2		2	2		0	1	
**Left bundle branch block, %**	3%	3%	-	2%	2%		8%	8%		0%	5%	
**Ventricular extrasystoles, n**	8	4	-	6	4	-	2	0		0	0	
**Ventricular extrasystoles, %**	5%	3%	-	6%	4%	-	8%	0%		0%	0%	
**QRS-interval, ms, median**	95	95	-	100	95	-	90	90	-	95	90	-
**QRS-interval, ms, IQR**	(20)	(25)	-	(20)	(20)	-	(20)	(20)	-	(18)	(18)	-
**R-amplitude in I, mV, median**	0.3	0.4	***	0.3	0.35	**	0.4	0.5	**	0.3	0.3	-
**R-amplitude in I, mV, IQR**	(0.29)	(0.25)	***	(0.25)	(0.25)	**	(0.3)	(0.4)	**	(0.25)	(0.18)	-
**R-amplitude in III, mV, median**	0.8	0.6	***	0.8	0.6	***	0.7	0.6	-	0.8	0.5	-
**R-amplitude in III, mV, IQR**	(0.64)	(0.5)	***	(0.65)	(0.53)	***	(0.4)	(0.55)	-	(0.68)	(0.43)	-
**R-amplitude in aVR, mV, median**	0.5	0.58	*	0.5	0.55	-	0.55	0.65	-	0.65	0.6	-
**R-amplitude in aVR, mV, IQR**	(0.3)	(0.35)	*	(0.28)	(0.35)	-	(0.25)	(0.2)	-	(0.3)	(0.3)	-
**R-amplitude in V1, mV, median**	0.3	0.2	***	0.33	0.2	***	0.2	0.15	*	0.2	0.1	-
**R-amplitude in V1, mV, IQR**	(0.35)	(0.25)	***	(0.3)	(0.26)	***	(0.25)	(0.21)	*	(0.1)	(0.1)	-
**R-amplitude in V2, mV, median**	0.3	0.25	**	0.3	0.25	-	0.25	0.25	-	0.38	0.3	*
**R-amplitude in V2, mV, IQR**	(0.3)	(0.25)	**	(0.4)	(0.31)	-	(0.31)	(0.15)	-	(0.15)	(0.25)	*
**R-amplitude in V6, mV, median**	0.8	0.8	*	0.8	0.78	*	0.85	0.8	-	0.85	0.85	-
**R-amplitude in V6, mV, IQR**	(0.5)	(0.5)	*	(0.45)	(0.54)	*	(0.5)	(0.35)	-	(0.43)	(0.58)	-
**S-amplitude in I, mV, median**	0.31	0.25	***	0.4	0.3	***	0.3	0.15	-	0.2	0.1	*
**S-amplitude in I, mV, IQR**	(0.35)	(0.25)	***	(0.35)	(0.25)	***	(0.3)	(0.2)	-	(0.23)	(0.2)	*
**S-amplitude in III, mV, median**	0.15	0.1	-	0.15	0.1	-	0.1	0.1	-	0.1	0	-
**S-amplitude in III, mV, IQR**	(0.3)	(0.3)	-	(0.3)	(0.3)	-	(0.2)	(0.2)	-	(0.55)	(0.35)	-
**S-amplitude in aVL, mV, median**	0.45	0.3	***	0.45	0.3	***	0.35	0.2	*	0.4	0.2	*

**S-amplitude in aVL, mV, IQR**	(0.5)	(0.45)	***	(0.5)	(0.45)	***	(0.2)	(0.35)	*	(0.63)	(0.33)	*
**S-amplitude in V1, mV, median**	0.3	0.4	***	0.25	0.35	***	0.3	0.45	*	0.5	0.5	-
**S-amplitude in V1, mV, IQR**	(0.35)	(0.4)	***	(0.3)	(0.43)	***	(0.35)	(0.35)	*	(0.43)	(0.35)	-
**S-amplitude in V3, mV, median**	0.7	0.6	*	0.6	0.6	-	0.85	0.6	-	0.95	0.85	-
**S-amplitude in V3, mV, IQR**	(0.64)	(0.54)	*	(0.6)	(0.5)	-	(0.45)	(0.45)	-	(0.58)	(0.6)	-
**S-amplitude in V4, mV, median**	0.65	0.5	***	0.65	0.55	***	0.6	0.45	*	0.5	0.6	-
**S-amplitude in V4, mV, IQR**	(0.65)	(0.5)	***	(0.6)	(0.5)	***	(0.65)	(0.4)	*	(0.55)	(0.53)	-
**S-amplitude in V5, mV, median**	0.5	0.4	***	0.55	0.45	***	0.4	0.3	**	0.4	0.3	-
**S-amplitude in V5, mV, IQR**	(0.45)	(0.4)	***	(0.5)	(0.45)	***	(0.6)	(0.3)	**	(0.48)	(0.38)	-
**S-amplitude in V6, mV, median**	0.3	0.2	***	0.3	0.25	***	0.2	0.1	**	0.1	0.05	*
**S-amplitude in V6, mV, IQR**	(0.4)	(0.29)	***	(0.4)	(0.28)	***	(0.35)	(0.15)	**	(0.25)	(0.18)	*
**R/S in V1, median**	1	0.4	***	1.2	0.5	**	0.8	0.3	*	0.3	0.3	-
**R/S in V1, IQR**	(2)	(0.8)	***	(2.2)	(0.9)	**	(1.2)	(0.7)	*	(0.3)	(0.2)	-
**R/S in V5, median**	1.7	2.4	***	1.7	2.2	***	1.9	3.2	-	2.1	3	**
**R/S in V5, IQR**	(2)	(2.7)	***	(1.6)	(2.4)	***	(2.4)	(3.1)	-	(2)	(3.6)	**
**R/S in V6, median**	2.4	3.3	***	2.3	3	***	2.6	6.3	**	3.8	5.8	*
**R/S in V6, IQR**	(2.6)	(4.4)	***	(2.6)	(3.6)	***	(3.3)	(9.4)	**	(6.2)	(6.5)	*
**R V1, 2 + S I, aVL - S V1, mV, median**	0.6	0.3	***	0.7	0.4	***	0.43	0.2	**	0.23	0.05	**
**R V1 ,2 + S I, aVL - S V1, mV, IQR**	(0.7)	(0.65)	***	(0.65)	(0.7)	***	(0.59)	(0.3)	**	(0.5)	(0.4)	**
**R V1 + S V5, V6, mV, median**	0.85	0.65	***	0.9	0.75	***	0.6	0.5	**	0.65	0.45	-
**R V1 + S V5, V6, mV, IQR**	(0.73)	(0.56)	***	(0.75)	(0.59)	***	(0.7)	(0.33)	**	(0.49)	(0.38)	-
**R-amplitude V1 (QRS < 120 ms), mV, median**	0.25	0.2	***	0.3	0.2	***	0.2	0.15	*	0.2	0.1	-
**R-amplitude V1 (QRS < 120 ms), mV, IQR**	(0.3)	(0.21)	***	(0.33)	(0.25)	***	(0.25)	(0.2)	*	(0.11)	(0.11)	-
**R peak time V1 (QRS < 120 ms), ms, median**	50	43	***	55	50	***	60	30	-	23	20	-
**R peak time V1 (QRS < 120 ms), ms, IQR**	(30)	(40)	***	(20)	(40)	***	(40)	(40)	-	(34)	(13)	-
**R V1, V2 + S I, V6 - S V1, mV, median**	0.6	0.28	***	0.7	0.4	***	0.5	0.08	**	0.1	-0.05	**
**R V1, V2 + S I, V6 - S V1, mV, IQR**	(0.89)	(0.7)	***	(0.75)	(0.75)	***	(0.66)	(0.43)	**	(0.25)	(0.55)	**

Annotation: SLI: Sokolow-Lyon-Index, IQR: interquartile range, mPAP: mean pulmonary artery pressure, -: $p > 0.05$, *: $p < 0.05$, **: $p < 0.01$, ***: $p < 0.001$

Table S19: Subgroups repolarisation parameters

	Entire cohort			mPAP ≥ 25			mPAP = 21-24			mPAP ≤ 20		
	**Baseline**	**Follow-up**	**p**	**Baseline**	**Follow-up**	**p**	**Baseline**	**Follow-up**	**p**	**Baseline**	**Follow-up**	**p**
**Longest T-wave, ms, median**	300	300	*	300	300	-	280	300	-	300	280	*
**Longest T-wave, ms, interquartile range**	(80)	(58)	*	(60)	(40)	-	(80)	(40)	-	(90)	(40)	*
**Shortest T-wave, ms, median**	150	140	-	150	140	-	140	150	-	140	140	-
**Shortest T-wave, ms, interquartile range**	(30)	(20)	-	(30)	(20)	-	(60)	(20)	-	(20)	(20)	-
**T-wave dispersion, ms, median**	140	140	-	140	140	-	140	140	-	160	140	-
**T-wave dispersion, ms, interquartile range**	(80)	(58)	-	(80)	(55)	-	(40)	(60)	-	(70)	(50)	-
**T-wave inversion in II, n**	46	19	***	33	12	***	6	3	-	4	1	-
**T-wave inversion in II, %**	(31%)	(13%)	***	(32%)	(12%)	***	(24%)	(12%)	-	(21%)	(5%)	-
**T-wave inversion in III, n**	73	49	***	54	37	**	9	7	-	7	2	*
**T-wave inversion in III, %**	(49%)	(33%)	***	(52%)	(36%)	**	(36%)	(28%)	-	(37%)	(11%)	*
**T-wave inversion in aVF, n**	61	26	***	47	20	***	6	3	-	5	0	
**T-wave inversion in aVF, %**	(41%)	(17%)	***	(46%)	(19%)	***	(24%)	(12%)	-	(26%)	(0%)	
**T-wave inversion in V1, n**	130	133	-	89	96	-	23	22	-	15	14	-
**T-wave inversion in V1, %**	(87%)	(89%)	-	(86%)	(93%)	-	(92%)	(88%)	-	(79%)	(74%)	-
**T-wave inversion in V2, n**	76	68	-	57	54	-	12	8	-	5	4	
**T-wave inversion in V2, %**	(51%)	(45%)	-	(55%)	(52%)	-	(48%)	(32%)	-	(26%)	(21%)	
**T-wave inversion in V3, n**	90	66	***	70	55	**	13	6	**	4	3	-
**T-wave inversion in V3, %**	(60%)	(44%)	***	(68%)	(53%)	**	(52%)	(24%)	**	(21%)	(16%)	-
**T-wave inversions in II, III, aVF and V1-V3, n**	21	9	*	15	7	-	3	1	-	1	0	
**T-wave inversions in II, III, aVF and V1-V3, %**	(14%)	(6%)	*	(15%)	(7%)	-	(12%)	(4%)	-	(5%)	(0%)	
**T-wave inversions in II, III and aVF, n**	46	18	***	33	12	***	6	3	-	4	0	
**T-wave inversions in II, III and aVF, %**	(31%)	(12%)	***	(32%)	(12%)	***	(24%)	(12%)	-	(21%)	(0%)	
**T-wave inversions in V1-V3, n**	68	53	*	53	45	-	11	4	*	2	3	-
**T-wave inversions in V1-V3, %**	(45%)	(35%)	*	(52%)	(44%)	-	(44%)	(16%)	*	(11%)	(16%)	-

Annotation: mPAP: mean pulmonary artery pressure, -: $p > 0.05$, *: $p < 0.05$, **: $p < 0.01$, ***: $p < 0.001$

Table S20: Subgroups general parameters

	Entire cohort			mPAP ≥ 25			mPAP = 21-24			mPAP ≤ 20		
	Baseline	Follow-up	p	Baseline	Follow-up	p	Baseline	Follow-up	p	Baseline	Follow-up	p
**Sinusrhythm, n**	148	143	*	101	98	-	25	25	/	19	17	/
**Sinusrhythm, %**	(99%)	(95%)	*	(98%)	(95%)	-	(100%)	(100%)	/	(100%)	(90%)	/
**Normocardia, n**	123	131	-	84	88	-	20	23	-	18	18	-
**Normocardia, %**	(82%)	(87%)	-	(82%)	(85%)	-	(80%)	(92%)	-	(95%)	(95%)	-
**Bradycardia, n**	3	9	-	2	6	-	1	2	-	0	1	/
**Bradycardia, %**	(2%)	(6%)	-	(2%)	(6%)	-	(4%)	(8%)	-	(0%)	(5%)	/
**Tachycardia, n**	24	10	**	17	9	-	4	0	/	1	0	/
**Tachycardia, %**	(16%)	(7%)	**	(17%)	(9%)	-	(16%)	(0%)	/	(5%)	(0%)	/
**First-degree AV block, n**	6	4	-	6	4	-	0	0	/	0	0	/
**First-degree AV block, %**	(4%)	(3%)	-	(6%)	(4%)	-	(0%)	(0%)	/	(0%)	(0%)	/
**Myocardial infarction, n**	6	6	/	5	5	/	1	1	/	0	0	/
**Myocardial infarction, %**	(4%)	(4%)	/	(5%)	(5%)	/	(4%)	(4%)	/	(0%)	(0%)	/
**Heart rate, bpm, median**	84	78	***	84	79	***	86	79	*	77	72	**
**Heart rate, bpm, IQR**	(21.8)	(19)	***	(20)	(19)	***	(20)	(16)	*	(19.5)	(11)	**
**PQ-interval, ms, median**	150	150	-	150	150	-	150	150	-	140	140	-
**PQ-interval, ms, IQR**	(29)	(25)	-	(20)	(23)	-	(28)	(28)	-	(30)	(30)	-
**PR-interval, ms, median**	160	145	/	160	170	/	120	175	/	150	120	/
**PR-interval, ms, IQR**	(20)	(41)	/	(8)	(30)	/	/	(25)	/	(30)	(10)	/
**QT-interval, ms, median**	390	380	***	400	380	**	380	370	*	400	370	-
**QT-interval, ms, IQR**	(70)	(40)	***	(80)	(50)	**	(50)	(40)	*	(40)	(30)	-
**QTc-interval (Bazett), ms, median**	454	432	***	459	436	***	452	412	***	432	399	**
**QTc-interval (Bazett), ms, IQR**	(85)	(44)	***	(88)	(44)	***	(79)	(35)	***	(45)	(30)	**
**QTc-interval (Fridericia), ms, median**	428	413	***	439	419	***	428	397	***	415	392	**
**QTc-interval (Fridericia), ms, IQR**	(77)	(43)	***	(92)	(42)	***	(49)	(35)	***	(38)	(30)	**
**QTc-interval (Hodges), ms, median**	428	414	***	435	418	***	428	399	***	416	391	**
**QTc-interval (Hodges), ms, IQR**	(69)	(39)	***	(78)	(35)	***	(53)	(33)	***	(32)	(27)	**

**QTc-interval (Framingham), ms, median**	390	380	***	400	380	**	380	370	*	400	370	-
**QTc-interval (Framingham), ms, IQR**	(70)	(40)	***	(80)	(50)	**	(50)	(40)	*	(40)	(30)	-

Annotation: mPAP: mean pulmonary artery pressure, IQR: interquartile range, -: p > 0.05, *: p < 0.05, **: p < 0.01, ***: p < 0.001

Table S21: Subgroups correlations between electrocardiographic and haemodynamic parameters - Baseline

		**Entire cohort**		**mPAP ≥ 25**		**mPAP = 21-24**		**mPAP ≤ 20**	
**Parameter 1**	**Parameter 2**	**r**	**p**	**r**	**p**	**r**	**p**	**r**	**p**
**Heart rate**	**mPAP**					0.405	< 0.05		
**Longest P-wave**	**mPAP**							-0.589	< 0.01
**PQ-interval**	**mPAP**							-0.502	< 0.05
**R-amplitude in I**	**mPAP**					-0.705	< 0.001		
**R-amplitude in III**	**mPAP**					0.451	< 0.05		
**R-amplitude in aVR**	**mPAP**					-0.637	< 0.001		
**R-amplitude in V1**	**mPAP**	0.473	< 0.001	0.369	< 0.001	0.427	< 0.05		
**S-amplitude in I**	**mPAP**	0.474	< 0.001	0.415	< 0.001	0.440	< 0.05		
**S-amplitude in aVL**	**mPAP**					0.404	< 0.05		
**S-amplitude in V1**	**mPAP**	-0.401	< 0.001			-0.643	< 0.001		
**S-amplitude in V4**	**mPAP**					0.705	< 0.001		
**S-amplitude in V5**	**mPAP**	0.344	< 0.001	0.330	< 0.001	0.468	< 0.05		
**S-amplitude in V6**	**mPAP**	0.419	< 0.001	0.314	< 0.01	0.566	< 0.01		
**R/S in V1**	**mPAP**	0.464	< 0.001			0.660	< 0.01		
**R/S in V6**	**mPAP**	-0.328	< 0.001						
**R/S in V5 : R/S in V1**	**mPAP**	-0.434	< 0.001			-0.524	< 0.05		
**(RI + SIII) - (SI + RIII)**	**mPAP**	-0.331	< 0.001			-0.670	< 0.001		
**R V1, V2 + S I, aVL - S V1**	**mPAP**	0.519	< 0.001	0.392	< 0.001	0.609	< 0.01		
**R V1, V2 + S I, V6 - S V1**	**mPAP**	0.524	< 0.001	0.420	< 0.001	0.555	< 0.01		
**R V1 + S V5, V6**	**mPAP**	0.471	< 0.001	0.411	< 0.001	0.604	< 0.01		
**R-amplitude in V1 (QRS < 120 ms)**	**mPAP**	0.458	< 0.001	0.408	< 0.001				
**R peak time in V1 (QRS < 120 ms)**	**mPAP**	0.324	< 0.001						
**QTc-interval (Bazett)**	**mPAP**	0.328	< .001			0.611	< 0.01		

**QTc-interval (Fridericia)**	**mPAP**					0.539	< 0.01		
**QTc-interval (Hodges)**	**mPAP**					0.595	< 0.01		
**Longest T-wave**	**mPAP**							0.468	< 0.05
**T-wave dispersion**	**mPAP**							0.466	< 0.05
**Heart rate**	**PVR**					0.459	< 0.05		
**Highest P-wave**	**PVR**	0.312	< 0.001	0.313	< 0.01				
**Longest P-wave**	**PVR**							-0.620	< 0.01
**R-amplitude in I**	**PVR**					-0.705	< 0.001	-0.631	< 0.01
**R-amplitude in aVR**	**PVR**					-0.734	< 0.001		
**R-amplitude in V1**	**PVR**	0.522	< 0.001	0.406	< 0.001	0.498	< 0.05	0.482	< 0.05
**S-amplitude in I**	**PVR**	0.372	< 0.001	0.340	< 0.001				
**S-amplitude in aVL**	**PVR**			0.308	< 0.01				
**S-amplitude in V1**	**PVR**	-0.409	< 0.001			-0.582	< 0.01		
**S-amplitude in V4**	**PVR**					0.672	< 0.001		
**S-amplitude in V5**	**PVR**	0.322	< 0.001			0.484	< 0.05		
**S-amplitude in V6**	**PVR**	0.441	< 0.001	0.333	< 0.001	0.605	< 0.01		
**R/S in V1**	**PVR**	0.518	< 0.001	0.394	< 0.001	0.702	< 0.001		
**R/S in V5**	**PVR**					-0.473	< 0.05		
**R/S in V6**	**PVR**	-0.384	< 0.001			-0.595	< 0.01		
**R/S in V5 : R/S in V1**	**PVR**	-0.501	< 0.001	-0.398	< 0.001	-0.620	< 0.01		
**R V1, V2 + S I, aVL - S V1**	**PVR**	0.491	< 0.001	0.400	< 0.001	0.498	< 0.05		
**R V1, V2 + S I, V6 - S V1**	**PVR**	0.516	< 0.001	0.434	< 0.001	0.527	< 0.01		
**R V1 + S V5, V6**	**PVR**	0.472	< 0.001	0.393	< 0.001	0.623	< 0.01		
**R-amplitude in V1 (QRS < 120 ms)**	**PVR**	0.496	< 0.001	0.413	< 0.001				
**R peak time in V1 (QRS < 120 ms)**	**PVR**	0.306	< 0.001						
**QT-interval**	**PVR**			0.326	< 0.01				
**QTc-interval (Bazett)**	**PVR**	0.447	< .001	0.398	< 0.001	0.569	< 0.01		
**QTc-interval (Fridericia)**	**PVR**	0.384	< .001	0.373	< 0.001	0.454	< 0.05		
**QTc-interval (Hodges)**	**PVR**	0.419	< 0.001	0.387	< 0.001	0.529	< 0.01		
**QTc-interval (Framingham)**	**PVR**			0.335	< 0.001				

**T-wave dispersion**	**PVR**					0.440	< 0.05		
**Heart rate**	**RAP**					0.468	< 0.05		
**P-wave amplitude in II**	**RAP**					0.610	< 0.01	0.497	< 0.05
**Highest P-wave**	**RAP**					0.610	< 0.01	0.497	< 0.05
**R-amplitude in I**	**RAP**					-0.519	< 0.01		
**R-amplitude in aVR**	**RAP**					-0.464	< 0.05	-0.510	< 0.05
**R-amplitude in V2**	**RAP**							-0.510	< 0.05
**S-amplitude in V3**	**RAP**	-0.311	< 0.001	-0.319	< 0.01				
**S-amplitude in V6**	**RAP**	0.315	< 0.001						
**R/S in V1**	**RAP**					0.458	< 0.05		
**R/S in V5**	**RAP**							-0.635	< 0.05
**R/S in V6**	**RAP**	-0.350	< 0.001	-0.312	< 0.01				
**(RI + SIII) - (SI + RIII)**	**RAP**					-0.519	< 0.01		
**R V1, V2 + S I, aVL - S V1**	**RAP**					0.601	< 0.01		
**R V1, V2 + S I, V6 - S V1**	**RAP**					0.549	< 0.01		
**R V1 + S V5, V6**	**RAP**					0.446	< 0.05		
**R-amplitude in V1 (QRS < 120 ms)**	**RAP**			0.313	< 0.01				
**R peak time in V1 (QRS < 120 ms)**	**RAP**	0.304	< 0.001	0.420	< 0.001				
**QTc-interval (Bazett)**	**RAP**					0.671	< 0.001		
**QTc-interval (Fridericia)**	**RAP**					0.591	< 0.01		
**QTc-interval (Hodges)**	**RAP**					0.655	< 0.001		
**R-amplitude in I**	**CO**							0.608	< 0.01
**R-amplitude in III**	**CO**					0.453	< 0.05		
**R-amplitude in aVR**	**CO**					0.508	< 0.05		
**R-amplitude in V1**	**CO**							-0.536	< 0.05
**R/S in V1**	**CO**					-0.506	< 0.05		
**R/S in V6**	**CO**					0.471	< 0.05		
**R/S in V5 : R/S in V1**	**CO**					0.472	< 0.05		
**QTc-interval (Hodges)**	**CO**			-0.300	< 0.01				

Annotation: r = 0.30 – 0.49, r = 0.50 – 0.69, r= 0.70 – 0.89, mPAP: mean pulmonary artery pressure, PVR: pulmonary vascular resistance, RAP: right atrial pressure, CO: cardiac output

Table S22: Subgroups correlations between electrocardiographic and haemodynamic parameters - Follow-up

		Entire cohort		mPAP ≥ 25		mPAP = 21-24		mPAP ≤ 20	
**Parameter 1**	**Parameter 2**	**r**	**p**	**r**	**p**	**r**	**p**	**r**	**p**
**PQ-interval**	**mPAP**							0.681	< 0.01
**R-amplitude in I**	**mPAP**					-0.484	< 0.05		
**R-amplitude in V2**	**mPAP**					-0.416	< 0.05		
**S-amplitude in I**	**mPAP**	0.356	< 0.001						
**S-amplitude in V1**	**mPAP**	-0.337	< 0.001						
**S-amplitude in V4**	**mPAP**					0.417	< 0.05		
**S-amplitude in V6**	**mPAP**	0.408	< 0.001						
**R/S in V1**	**mPAP**	0.350	< 0.001						
**R/S in V5 : R/S in V1**	**mPAP**	-0.359	< 0.001	-0.341	< 0.01				
**R V1, V2 + S I, aVL - S V1**	**mPAP**	0.407	< 0.001						
**R V1, V2 + S I, V6 - S V1**	**mPAP**	0.423	< 0.001						
**R V1 + S V5, V6**	**mPAP**	0.342	< 0.001						
**QTc-interval (Bazett)**	**mPAP**	0.322	< .001						
**QTc-interval (Hodges)**	**mPAP**	0.318	< 0.001						
**R-amplitude in V2**	**PVR**							-0.523	< 0.05
**S-amplitude in I**	**PVR**	0.329	< 0.001						
**S-amplitude in V1**	**PVR**	-0.344	< 0.001						
**S-amplitude in V6**	**PVR**	0.394	< 0.001						
**R/S in V1**	**PVR**	0.335	< 0.001						
**R/S in V6**	**PVR**	-0.305	< 0.001						
**R/S in V5 : R/S in V1**	**PVR**	-0.336	< 0.001	-0.324	< 0.01				
**R V1, V2 + S I, aVL - S V1**	**PVR**	0.372	< 0.001						
**R V1, V2 + S I, V6 - S V1**	**PVR**	0.393	< 0.001						
**QTc-interval (Bazett)**	**PVR**	0.357	< 0.001						
**QTc-interval (Fridericia)**	**PVR**	0.315	< 0.001						
**Longest T-wave**	**PVR**					0.436	< 0.05		

**T-wave dispersion**	**PVR**	0.428	< 0.05		
**PQ-interval**	**RAP**			0.672	< 0.01
**R-amplitude in I**	**RAP**	0.476	< 0.05		
**R-amplitude in III**	**RAP**	0.480	< 0.05		
**R-amplitude in aVR**	**RAP**	0.733	< 0.001		
**S-amplitude in V3**	**RAP**	0.422	< 0.05		
**S-amplitude in III**	**CO**	-0.436	< 0.05		
**R/S in V1**	**CO**	-0.494	< 0.05	0.560	< 0.05
**R/S in V5 : R/S in V1**	**CO**	0.473	< 0.05		
**Longest T-wave**	**CO**	-0.425	< 0.05		
**T-wave dispersion**	**CO**	-0.417	< 0.05		

Annotation: r = 0.30 – 0.49, r = 0.50 – 0.69, r= 0.70 – 0.89, mPAP: mean pulmonary artery pressure, PVR: pulmonary vascular resistance, RAP: right atrial pressure, CO: cardiac output

Table S23: Subgroups correlations between changes in electrocardiographic and changes in haemodynamic parameters

		**Entire cohort**		**mPAP ≥ 25**		**mPAP = 21-24**		**mPAP ≤ 20**	
**Parameter 1**	**Parameter 2**	**r**	**p**	**r**	**p**	**r**	**p**	**r**	**p**
**Heart rate**	**mPAP**					0.406	< 0.05	0.525	< 0.05
**P-wave amplitude in II**	**mPAP**	0.379	< 0.001	0.366	< 0.001			0.500	< 0.05
**Highest P-wave**	**mPAP**	0.407	< 0.001	0.407	< 0.001			0.500	< 0.05
**R-amplitude in III**	**mPAP**							0.472	< 0.05
**R-amplitude in aVR**	**mPAP**					-0.525	< 0.01		
**R-amplitude in V2**	**mPAP**	0.383	< 0.001	0.414	< 0.001			0.516	< 0.05
**R-amplitude in V6**	**mPAP**					-0.464	< 0.05		
**S-amplitude in V1**	**mPAP**					-0.745	< 0.001		
**S-amplitude in V4**	**mPAP**					0.514	< 0.01		
**S-amplitude in V6**	**mPAP**					0.451	< 0.05		
**R/S in V1**	**mPAP**					0.715	< 0.001		
**R/S in V6**	**mPAP**	-0.381	< 0.001	-0.403	< 0.001				
**R V1, V2 + S I, aVL - S V1**	**mPAP**	0.488	< 0.001	0.415	< 0.001	0.817	< 0.001		
**R V1, V2 + S I, V6 - S V1**	**mPAP**	0.482	< 0.001	0.426	< 0.001	0.749	< 0.001		

**R-amplitude in V1 (QRS < 120 ms)**	**mPAP**			0.309	< 0.01				
**QT-interval**	**mPAP**					0.426	< 0.05		
**QTc-interval (Bazett)**	**mPAP**	0.302	< 0.001			0.681	< 0.001		
**QTc-interval (Fridericia)**	**mPAP**					0.639	< 0.001		
**QTc-interval (Hodges)**	**mPAP**					0.673	< 0.001		
**QTc-interval (Framingham)**	**mPAP**					0.455	< 0.05		
**Heart rate**	**PVR**					0.402	< 0.05	0.512	< 0.05
**P-wave amplitude in II**	**PVR**	0.339	< 0.001	0.359	< 0.001				
**Highest P-wave**	**PVR**	0.397	< 0.001	0.442	< 0.001				
**Shortest P-wave**	**PVR**			0.339	< 0.001				
**R-amplitude in V1**	**PVR**	0.422	< 0.001			0.541	< 0.01		
**R-amplitude in V2**	**PVR**	0.335	< 0.001	0.388	< 0.001				
**R-amplitude in V6**	**PVR**	-0.387	< 0.001	-0.421	< 0.001	-0.478	< 0.05		
**S-amplitude in III**	**PVR**					0.560	< 0.01		
**S-amplitude in V1**	**PVR**					-0.655	< 0.001		
**S-amplitude in V3**	**PVR**							0.526	< 0.05
**S-amplitude in V4**	**PVR**	0.309	< 0.001			0.664	< 0.001		
**S-amplitude in V5**	**PVR**					0.405	< 0.05		
**S-amplitude in V6**	**PVR**	0.322	< 0.001			0.577	< 0.01		
**R/S in V1**	**PVR**	0.454	< 0.001	0.442	< 0.001	0.688	< 0.001		
**R/S in V5**	**PVR**	-0.305	< 0.001			-0.465	< 0.05		
**R/S in V6**	**PVR**	-0.419	< 0.001	-0.443	< 0.001				
**R/S in V5 : R/S in V1**	**PVR**	-0.357	< 0.001	-0.312	< 0.01	-0.608	< 0.01		
**R V1, V2 + S I, aVL - S V1**	**PVR**	0.418	< 0.001	0.394	< 0.001	0.601	< 0.01		
**R V1, V2 + S I, V6 - S V1**	**PVR**	0.494	< 0.001	0.481	< 0.001	0.697	< 0.001		
**R V1 + S V5, V6**	**PVR**	0.360	< 0.001	0.335	< 0.01	0.556	< 0.01		
**R-amplitude in V1 (QRS < 120 ms)**	**PVR**	0.493	< 0.001	0.524	< 0.001	0.501	< 0.05		
**R peak time in V1 (QRS < 120 ms)**	**PVR**	0.306	< 0.001			0.444	< 0.05		
**QTc-interval (Bazett)**	**PVR**			0.341	< 0.001	0.595	< 0.01		
**QTc-interval (Fridericia)**	**PVR**					0.548	< 0.01		

**QTc-interval (Hodges)**	**PVR**					0.594	< 0.01		
**P-wave amplitude in II**	**RAP**					0.492	< 0.05		
**Highest P-wave**	**RAP**					0.492	< 0.05		
**QRS-interval**	**RAP**							-0.590	< 0.05
**S-amplitude in aVL**	**RAP**							-0.523	< 0.05
**S-amplitude in V4**	**RAP**					0.476	< 0.05		
**R/S in V1**	**RAP**					0.586	< 0.05		
**R V1, V2 + S I, aVL - S V1**	**RAP**					0.447	< 0.05		
**R V1, V2 + S I, V6 - S V1**	**RAP**					0.547	< 0.01		
**QTc-interval (Bazett)**	**RAP**					0.590	< 0.01		
**QTc-interval (Fridericia)**	**RAP**					0.456	< 0.05		
**QTc-interval (Hodges)**	**RAP**					0.490	< 0.05		
**Heart rate**	**CO**							-0.497	< 0.05
**R-amplitude in V1**	**CO**					-0.447	< 0.05		
**R-amplitude in V6**	**CO**	0.318	< 0.001	0.342	< 0.001				
**S-amplitude in III**	**CO**					-0.590	< 0.01		
**S-amplitude in V6**	**CO**					-0.552	< 0.01		
**R/S in V1**	**CO**	-0.335	< 0.001	-0.374	< 0.01				
**R/S in V5 : R/S in V1**	**CO**					0.529	< 0.05		
**R peak time in V1 (QRS < 120 ms)**	**CO**					-0.496	< 0.05		
**QT-interval**	**CO**							0.554	< 0.05
**QTc-interval (Framingham)**	**CO**							0.515	< 0.05

Annotation: r = 0.30 – 0.49, r = 0.50 – 0.69, r= 0.70 – 0.89, mPAP: mean pulmonary artery pressure, PVR: pulmonary vascular resistance, RAP: right atrial pressure, CO: cardiac output
